# Synthesis of 1-(2-Fluorophenyl)pyrazoles by 1,3-Dipolar Cycloaddition of the Corresponding Sydnones

**DOI:** 10.3390/molecules26123693

**Published:** 2021-06-17

**Authors:** Denisa Dumitrescu, Sergiu Shova, Constantin Draghici, Marcel Mirel Popa, Florea Dumitrascu

**Affiliations:** 1 Faculty of Pharmacy, Ovidius University Constanta, Str. Cpt. Av. Al. Serbanescu, Campus Corp C, 900470 Constanta, Romania; denisa.dumitrescu2014@gmail.com; 2 CEEC Institute, Ningbo University of Technology, No. 201 Fenghua Road, Ningbo 315021, China; shova@icmpp.ro; 3 Department of Inorganic Polymers, “Petru Poni” Institute for Macromolecular Chemistry, Romanian Academy, Aleea Grigore Ghica Voda, 41A, 700487 Iasi, Romania; 4 Center of Organic Chemistry ‘‘C. D. Nenitzescu’’, Roumanian Academy, Spl Independentei 202B, 060023 Bucharest, Romania; cstdrag@yahoo.com

**Keywords:** sydnone, 1,3-dipolar cycloaddition, pyrazole, X-ray diffraction, halogen bonding

## Abstract

3-Arylsydnones bearing fluorine and bromine atoms on the benzene ring were synthesized from *N*-nitroso-2-fluorophenylglycines and characterized by NMR spectroscopy. These were employed further in synthesis of the corresponding 1-(2-fluorophenyl)pyrazoles by 1,3-dipolar cycloaddition reaction with dimethyl acetylenedicarboxylate (DMAD) as activated dipolarophile. The sydnones as reaction intermediates were characterized by single crystal X-ray diffraction analysis showing interesting features such as halogen bonding as an important interaction in modeling the crystal structure.

## 1. Introduction

**Figure 1:**
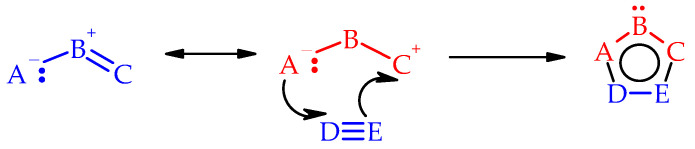
Schematic representation of 1,3-dipolar cycloaddition reaction between a formal 1,3-dipole and an (acetylenic) dipolarophile.

The 1,3-dipolar cycloadditions reactions [1], also known as “Huisgen reactions” [2], involving 1,3-dipoles from the class of *N*-ylides [3,4,5,6], mesoionic compounds such as munchnones [7,8] and sydnones [9,10] and many others [11,12], have been intensively studied in obtaining a wide range of five membered heterocycles (Figure 1) [13].

Sydnones are mesoionic compounds with interesting properties and increased synthetic utility as synthons for creating five membered heterocycles [14,15,16,17,18,19,20,21,22,23,24]. The important biological properties of sydnones were reviewed recently [25]. On the other hand, 1-phenylpyrazoles generated by 1,3-dipolar cycloaddition between sydnones as dipoles and dimethylacetylene dicarboxylate as alkyne dipolarophile are also important bioactive scaffolds [26,27] 

Attaching halogenated atoms to organic frameworks could improve the bioavailability of such compounds [28,29,30,31,32,33,34]. Introducing fluorine atoms on a small molecule framework dramatically influences its properties regarding the interaction with specific target enzymes from simple dipole–dipole interactions to the most newly investigated halogen bonds [29,30,31,32,33]. Moreover, (2-fluorophenyl)pyrazoles [35,36] were reported to present anticancer activity [37] and are important ligands for organometallic applications [38]. We have shown also that halogenated pyrazoles are important tools for studying the halogen bonding propensity [39,40] and it was interesting to investigate if the fluorine atom could also play a role among the intermolecular interactions.

Given our interest in the chemistry of nitrogen containing heterocycles [41,42,43], we present herein the synthesis of new (2-fluorophenyl)pyrazoles also bearing bromine atoms, starting from the corresponding sydnones and in presence of DMAD as dipolarophile. The synthesis is straightforward and implies usual conditions. 

## 2. Results and Discussion

### 2.1. Synthesis and Spectral Analysis

Sydnones are accessible tools in the synthesis of pyrazoles and thus they were employed successfully to obtain a large diversity of such compounds. At their turn, the sydnones are synthesized by the nitrosation and subsequent cyclization of *N*-phenyl glycines in acetic anhydride [44].

**Scheme 1:**
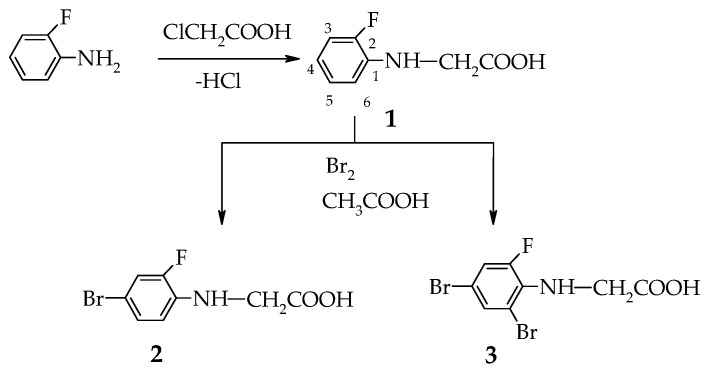
The synthesis of the starting halogenated *N*-phenylglycines.

The first step was the obtaining of *N*-phenylglycine **1** by reacting 2-fluoroaniline with 2-chloroacetic acid [45]. Compound **1** was then brominated using Br_2_ in glacial acetic acid as solvent to obtain the new polyhalogenated *N*-phenylglycines **2** and **3**. The bromination reactions worked with 78% and 90% yield, respectively (Scheme 1).

**Figure 2:**
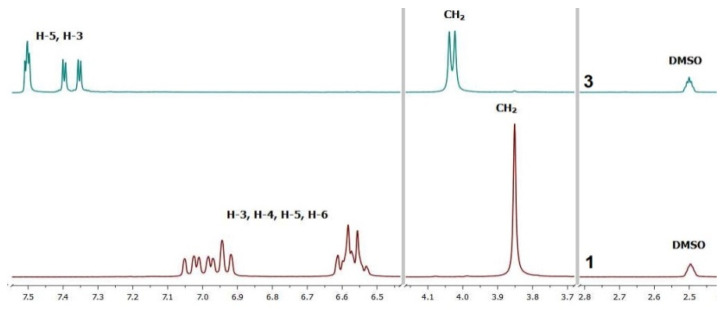
^1^H NMR spectra of compounds **1** and **3** showing the aromatic and aliphatic relevant regions.

The structure of the phenylglycines **1**–**3** was assigned on the basis of NMR spectroscopy. Both ^1^H and ^13^C spectra are in agreement with the proposed structures. The heteronuclear coupling ^19^F-^1^H induces specific multiplet signals. The CH_2_ hydrogens appear in the range 3.85–4.03 ppm with the interesting observation that for the compound **3** the signal is split into a doublet with *J* = 4.7 Hz due to the heteronuclear spin–spin long range coupling with the fluorine atom in the benzene ring (Figure 2). For the other two compounds, the coupling could not be observed. This could be an effect of the hindered rotation about the C-N bond due to the bromine atom in the *ortho* position. The ^13^C NMR spectra are also in good agreement with the structure of the compounds **1**–**3**. The main signals and the multiplicities raised by the ^19^F-^13^C heteronuclear spin–spin coupling are presented in Table 1. For the compound **3**, the same observation was made for ^13^C spectrum as for the ^1^H such that the signal of the CH_2_ carbon atom appears as a doublet at 45.5 ppm with *J* = 9.2 Hz. Interestingly, the carbon atom in the C=O group signal appears as a doublet at 172.3 with *J*_19F-13C_ = 2.1 Hz.

**Scheme 2:**
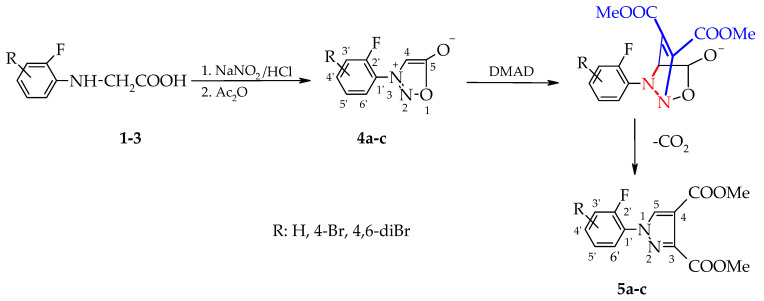
The synthesis of halogenated sydnones **4a**–**c** and the corresponding pyrazoles **5a**–**c**.

The *N*-phenylglycines **1**–**3** were employed in the synthesis of 3-arylsydnones **4a**–**c** by an improved method, which implies the in situ nitrosation reaction and further cyclization with Ac_2_O according to Scheme 2.

The compounds **4a**–**c** were also characterized by NMR spectroscopy. The main ^1^H NMR features are given by the specific multiplicities of the signals of the hydrogen atoms in the benzene ring owing to the ^1^F-^1^H spin–spin coupling. The signal of the H-4 sydnone hydrogen appears in the range 6.53–6.81 ppm. For the compounds **4a**,**b** multiplicity of the signal of H-4 is a doublet with *J* = 2.3 Hz. For the compound **4c** the analogous signal for H-4 appears as a sharp singlet due to the hindered C-N rotation induced by the bulky bromine atom in the *ortho* position of the phenyl ring with respect to the sydnone moiety. The main characteristic signals in the ^13^C NMR spectra are presented in Table 1. Similarly to the observations made on the ^1^H NMR spectra, the signal of the sydnone CH appears in the range 97.0–98.1 ppm with a multiplicity of doublet for compounds **4a**,**b** with *J* ~ 0.7 Hz, which is not observed for the compound **4c**. Another interesting aspect is the heteronuclear ^19^F-^13^C coupling constant observed in the case of C-6′, which is very small, close to 1 Hz, knowing that values for a *meta* coupling should be in the range 4–5 Hz. All the other coupling constants are as expected.

The 1-arylpyrazoles **5a**–**c** were obtained by 1,3-dipolar cycloaddition of the sydnones **4a**–**c** with dimethyl acetylenedicarboxylate (DMAD) as electron deficient alkyne in toluene or xylene as solvent (Scheme 2). The new compounds were obtained in good yields and were also characterized by NMR spectroscopy. The main characteristics of the ^1^H NMR spectra are the signals of the pyrazole hydrogen H-5, which appears as a doublet with *J* = 2.5 Hz at around 8.43 ppm for compounds **5a**,**b**, whereas for compound **5c** it appears as a singlet slightly shielded at 8.07 ppm. All the other NMR signals are in accordance with the structure and the multiplicities are influenced by the ^19^F-^1^H heteronuclear spin–spin coupling. The ^13^C NMR signals are shown also in Table 1. The carbon atom C-5 appears as a doublet with *J* = 10 Hz for **5a**,**b** whereas for **5c** it appears as a sharp singlet due to the hindered rotation about C-N bond which minimizes the chances of trough space coupling between the C5 or H5 and the fluorine atom. The small value of the *J*_19F-13C_ ~ 1 Hz is observed also in the case of pyrazoles.

### 2.2. X-ray Diffraction Analysis

The solid state structures of the synthesized compounds have been determined using single-crystal X-ray diffraction method and their crystallographic parameters are shown in Table 2.

**Figure 3:**
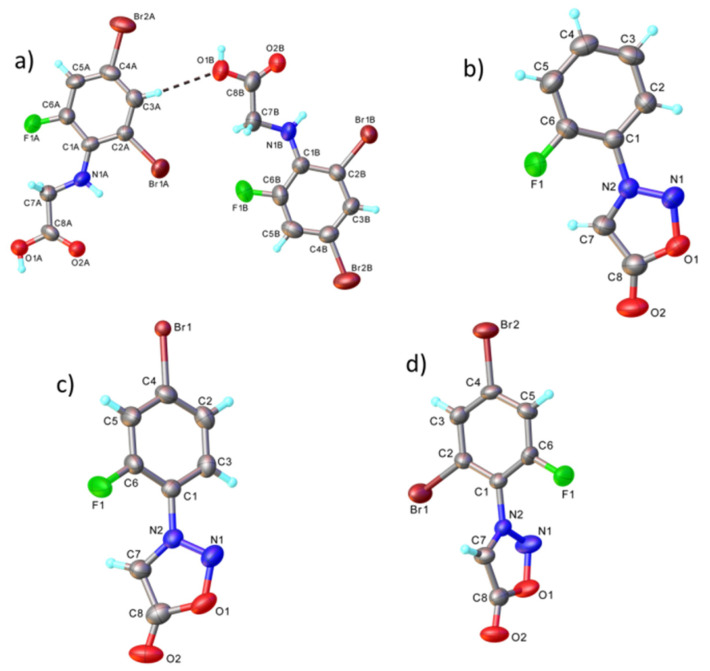
View of the asymmetric part of the unit cell in the crystal structure of compounds **3** (**a**), **4a** (**b**), **4b** (**c**) and **4c** (**d**) with atom labeling scheme and thermal ellipsoids at 50% level. H-bonds parameters for compound **3**: C3A-H···O1B [O3A-H 0.93 Å, H···O1B 2.53 Å, O3A···O1B 3.430(7) Å, ∠C3AHO1B 162.2°].

According to X-ray crystallography, the investigated compounds present a molecular crystal structure that is built-up from molecular units, as depicted in Figure 3. The asymmetric part of the unit cell in the crystal structure of **3** comprises two crystallographic independent but chemically identical molecules, denoted below as **A** and **B** components. The analysis of the molecular structure has revealed the molecule **3** to exhibit a planar configuration (see Table S1). On the contrary, due to *ortho*-substitution in aromatic rings, the molecules **4a**, **4b** and **4c** are essentially non-planar (Table S2). The dihedral angle formed by two cyclic fragments is of 35.61(9)°, 50.2(1)° and 78.5(1)° for **4a**, **4b**, and **4c**, respectively.

**Figure 4:**
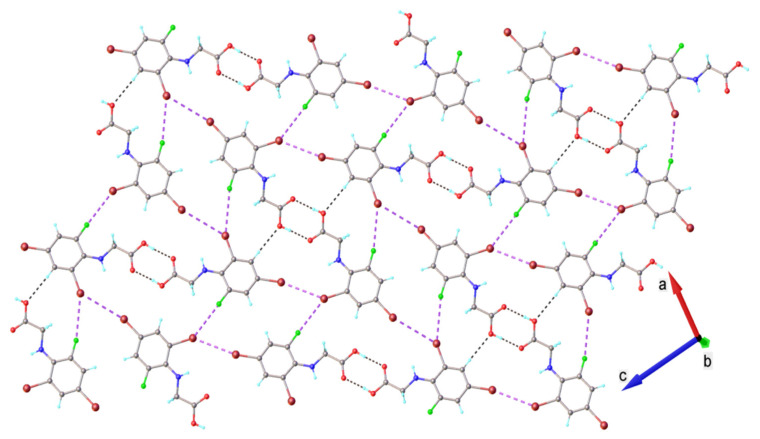
View of two-dimensional supramolecular layer showing the hydrogen bonding and halogen···halogen short contacts in the crystal structure **3**. Black and purple dashed lines are used for H-bonds and Br···Br and Br···F contacts, respectively. **H-bonds parameters**: O1A-H···O2A [O1A-H 0.82 Å, H···O2A 1.82 Å, O1A···O2A(1 − *x*, 1 − *y*, 2 − *z*) 2.633(5) Å, ∠O1AHO2A 174.4°]; O1B-H···O2B [O1B-H 0.82 Å, H···O2B 1.82 Å, O1B···O2B(−*x*, −*y*, −*z*) 2.629(5) Å, ∠O1BHO2B 169.3°]; C3B-H···O1A [O3B-H 0.93 Å, H···O1A 2.56 Å, C3B···O1A(1 + *x*, *y*, *z* − 1) 3.409(6) Å, ∠O3BHO1A 152.0°]; **Hal···Hal short contacts**: C2A-Br1A···Br1A-C2A(1 − *x*, 1 − *y*, 1 − *z*) [Br1A···Br1A’ 3.699(1) Å, ∠C2A-Br1A···Br1A’ 151.1(2)°]; C2B-Br1B···Br1B-C2B(−*x*, −*y*, −*z*) [Br1B···Br1B’ 3.614(1) Å, ∠C2B-Br1B···Br1B’ 143.5(2)°]. C6A-F1A···Br1B-C2B(*x* − 1, *y*, 1 + *z*) [F1A···Br1B’ 3.365(3) Å, ∠C6A-F1A···Br1B’ 145.8(4)°, C2B-Br1B···F1A’ 143.0(2)°]; C6B-F1B···Br1A-C2A [F1B···Br1A 3.456(3) Å, ∠C6B-F1B···Br1A 157.1(4)°, C2A-Br1A···F1B 143.6(2)°].

The further analysis of the crystal structure has shown the important role of hydrogen bonding, π-π stacking and homo- and hetero-halogen X···X (Br, F) interactions, which determine the formation of 2D supramolecular architecture as the main packing motif for the investigated compounds. Thus, the both crystallographically independent carboxylic groups in compound **3** are involved into the formation of the stable cyclic O-H···O H-bonded syntons. The system of intermolecular interaction is completed by the short Br···Br and F···Br contacts in adjacent molecules. These interactions are responsible for the supramolecular aggregation of the H-bonded synthons into two-dimensional supramolecular layers, as shown in Figure 4. It should be noted that, due to the steric effect of adjacent oxygen and bromine atoms, N-H groups are not involved in the intermolecular hydrogen bonding. 

**Figure 5:**
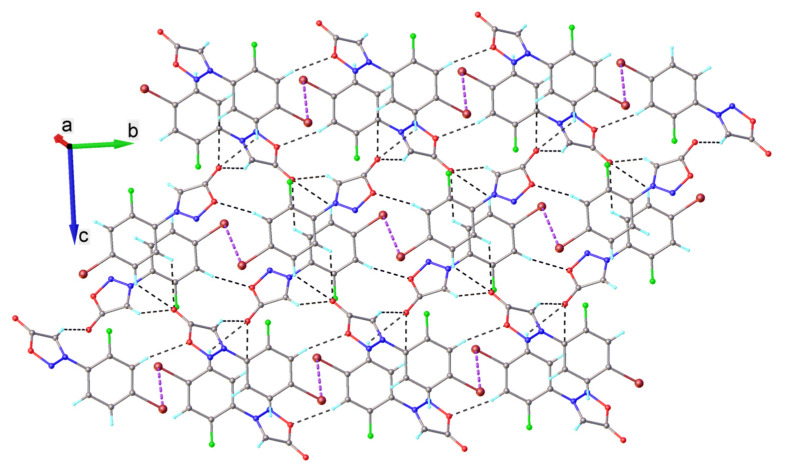
2D suparmolecular layer in the crystal structure of **4b**. Black and purple dashed lines are used for H-bonds and Br···Br contacts, respectively. H-bonds parameters: C3-H···O2 [O3-H 0.93 Å, H···O2 2.62 Å, C3···O2(*x*, 0.5 − *y*, *z* − 0.5) 3.260(3) Å, ∠O3HO2 126.3°]; C5-H···O1 [O5-H 0.93 Å, H···O1 2.62 Å, C5···O1(*x*, 1 + *y*, *z*) 3.389(3) Å, ∠O5HO1 160.7°]; C7-H···O2 [O7-H 0.93 Å, H···O2 2.35 Å, C7···O2(*x*, 1 + *y*, *z*) 3.186(3) Å, ∠O7HO2 149.7°].

A view of 2D organic network in the crystal structure of **4b** is shown in Figure 5. This supramolecular architecture is stabilized via weak intermolecular C-H···O H-bonds, where both oxygen atoms acts as acceptor of protons. The Br···Br short contacts did not present the geometrical requirements for halogen–halogen bonding pink dashed line. The crystal structure of compounds **3** and **4b** is similar. It consists from the parallel packing of 2D layers driven by π-π stacking interactions between aromatic rings belonging to adjacent layers, which are evidenced by the short centroid-to centroid distances of 3.7568(2) Å. As a result, the crystal structure of compounds **3** and **4b** can be characterized as a 3D supramolecular network. A view of the packing diagram for compounds **3** and **4b** is shown in Figure S1 (Supplementary Materials).

Compared to the compounds **3** and **4b**, the crystal structure of compounds **4a** and **4c** is built-up from the parallel packing of the discrete weakly interacting two-dimensional supramolecular double-layers, as shown in Figure S2.

**Figure 6:**
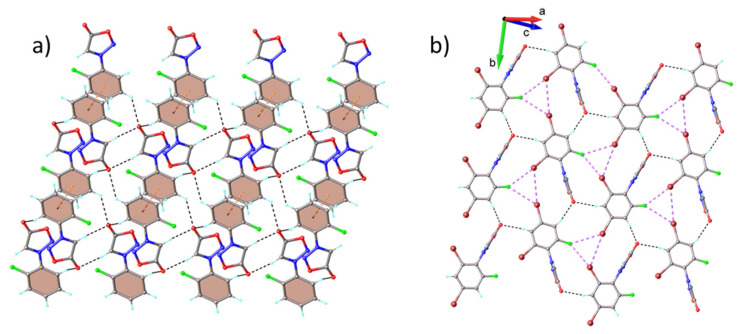
View of double layer network in the crystal of **4a**, showing the role of hydrogen bonding and π-π stacking (**a**), the system of intermolecular interactions in 2D supramolecular unit of **4c** (**b**). Black and purple dashed lines are used for H-bonds and Br···Br and Br···F contacts close to the limit of the vdW radii. Centroid-to-centroid distances at 3.6267(3) Å are shown in dashed-orange lines. **H-bonds parameters for 4a**: C2-H···O2 [C2-H 0.93 Å, H···O2 2.61 Å, C2···O2(−*x*, 1 − *y*,1 + *z*) 3.364(3) Å, ∠C2HO2 138.0°]; C3-H···O2 [C3-H 0.93 Å, H···O2 2.59 Å, C3···O2(1 + *x*, *y*, 1 + *z*) 3.251(3) Å, ∠C3HO2 128.6°]; C7-H···O2 [C7-H 0.93 Å, H···O2 2.59 Å, C7···O2(−1 − *x*, 1 − *y*, −*z*) 3.313(3) Å, ∠C7HO2 152.2°]; **H-bonds parameters for 4b**: C3-H···O2 [C3-H 0.93 Å, H···O2 2.54 Å, C3···O2(−0.5 + *x*, 0.5 − *y*, −0.5 + *z*) 3.422(3) Å, ∠C3HO2 159.3°]; C5-H···O2 [C3-H 0.93 Å, H···O2 2.69 Å, C5···O2(*x*, −1 + *y*,1 *z*) 3.380(5) Å, ∠C5HO2 131.2°]; C7-H···O2 [C7-H 0.93 Å, H···O2 2.46 Å, C7···O2(1.5 − *x*, −0.5 + *y*, 1.5 − *z*) 3.031(5) Å, ∠C7HO2 119.5°]; **Hal···Hal short contacts for 4c**: C4-Br2···Br1-C2(*x*, *y* − 1, *z*) [Br2···Br1’ 3.7637(7) Å, ∠C2-Br2···B1r’-C2 166.5(1)°, ∠C6-F1···Br1-C4 125.9(1)Å]; C6-F1···Br1-C2(0.5 + *x*, −0.5 + *y*, 0.5 + *z*) [F1···Br1’ 3.269(2) Å, ∠C6-F1···Br1’-C2 141.3(2)°, ∠C2-Br1···F1-C6 157.9(2)Å]; C6-F1···Br2-C4(0.5 + *x*, −0.5 + *y*, 0.5 + *z*).

The double layer in the crystal of **4a** is formed from the molecular units linked through C-H···O H-bonds and stacking interactions (see Figure 6a), while in the crystal structure of **4c**, is formed from two symmetric 2D supramolecular units, where the neutral molecules are self-assembled through C-H···O hydrogen bonding, as depicted in Figure 6b. The system of intermolecular interaction in **4c** is completed by F···Br and Br···Br short contacts (see Figure 6b).

### 2.3. Hirshfeld Analysis

For the representative compounds Hirshfeld analysis as implemented in CrystalExplorer [46] confirm the supra-molecular interactions and also show in a suggestive way the important crystal arrangement driving forces.

**Figure 7:**
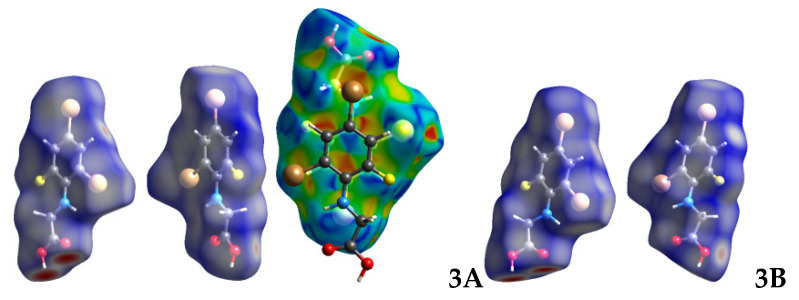
Hirshfeld surface of the two independent molecules of **3**. For **3A**, the shape index mode of the Hirshfeld surface shows the complementary spots corresponding to the π-π stacking.

Compound **3**. For the acid **3** it is important to note the existence of the two independent molecules **3A** and **3B**. It appears that the O···H bond involving the carboxylic acid groups are established between the same kind of molecular entities forming dimers. These dimers are connected together through one O···H bond involving H-3′ and the oxygen in the hydroxyl atom of the acid of an adjacent molecule and halogen bonds involving Br···Br and Br···F (at the limit of the sum of the vdW radii) contacts as described in Figure 3 from the X-ray diffraction chapter. All these interactions form 2D sheets, which are connected through π···π stacking between two similar molecules and presumably lone-pair···π between molecules of type **3B**. Figure 7 shows the Hirshfeld surfaces of the two independent molecules of **3**, and the shape index mode showing the π-π interactions in molecules **3A**.

**Figure 8:**
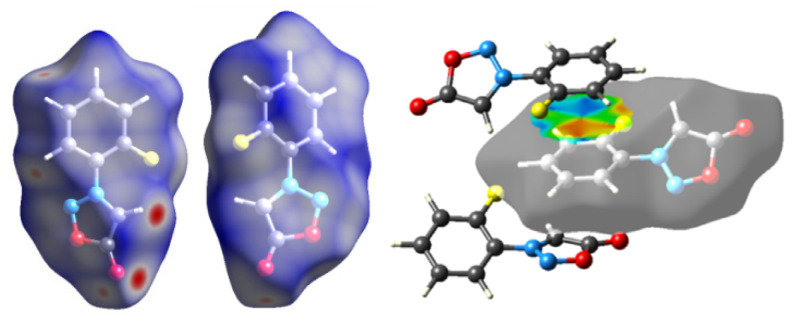
Hirshfeld surface of **4a** showing the main contacts for the O···H bonds. π-π stacking is also highlighted.

Compound **4a**. The sydnone **4a** does not have any halogen atom attached besides the fluorine atom. This suggests that the strong intermolecular forces are C-H···O hydrogen bonding, implying the exocyclic carbonyl oxygen of the sydnone. The red spots on the Hirshfeld surface depict the contact places for the C-H···O interactions (Figure 8). 

**Figure 9:**
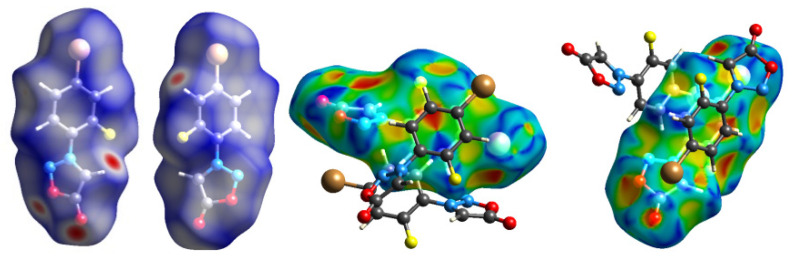
Hirshfeld surface of **4b** showing the main contacts for the O···H bonds. Shape index mode of the Hirshfeld surface shows the complementary spots corresponding to the π···π stacking.

Compound **4b**. Adding a Br atom in the *para* position of the phenyl ring in respect to the sydnone did not change dramatically the spatial arrangement of the molecules. The main contacts observed also from the Hirshfeld surface are O···H (Figure 9) bonds involving the sydnone moiety and H-3′ atom between the two Br atoms (red spots). All these interactions form stair-like arrangements which are held together by π···π interactions. It appears that Br atom is not involved in any halogen bonding type contact besides the hydrogen bonds in which it is involved.

Compound **4c**. The addition of the second Br atom in the 6′ position in respect to the sydnone ring preserved the role of the sydnone moiety in forming hydrogen bonds by its oxygen and hydrogen atoms and somehow similar stair-like pattern as for **4b** was observed, held together by π···π bonds.

**Figure 10:**
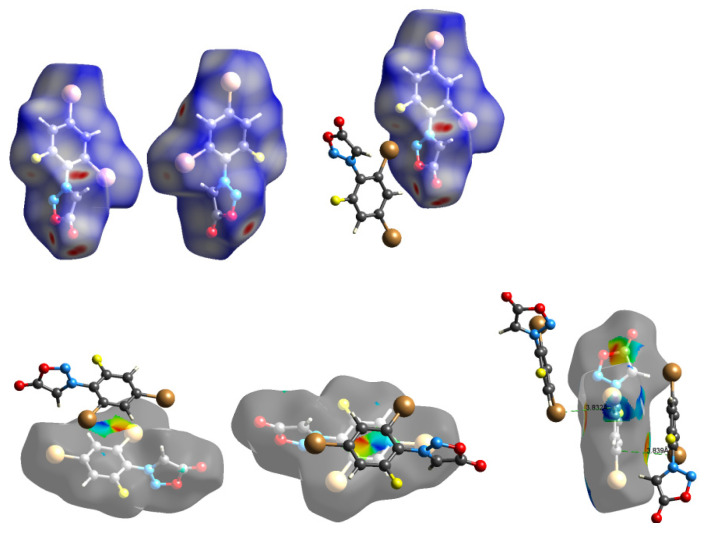
Hirshfeld surface of **4c** showing the main contacts for the O···H bonds. Shape index mode of the Hirshfeld surface shows the complementary spots corresponding to the π···π stacking.

Layers are formed in the plane of the phenyl atoms by F···Br, Br···Br and Br···Syd and H-3′···O=C (Syd). These layers are interconnected by O···H contacts involving the sydnone moiety, π···π interactions between the phenyl rings on one part and Br···π of type lone pair···π on the other face of the phenyl ring (Figure 10).

## 3. Materials and Methods

Melting points were determined on a Boëtius hot plate microscope (Carl Zeiss, Jena, Germany and are uncorrected. The elemental analysis was carried out on a COSTECH Instruments EAS32 apparatus (Costech Analytical Technologies, Valencia, CA, USA). The NMR spectra were recorded on a Varian Gemini 300 BB instrument (Varian, Palo Alto, CA, USA), operating at 300 MHz for ^1^H-NMR and 75 MHz for ^13^C-NMR or Bruker Avance Neo (Bruker, Billerica, MA, USA) operating at 400 MHz and 125 MHz for compound **4c**. Supplementary evidence was given by HETCOR and COSY experiments. 

X-ray diffraction measurements were carried out with a Rigaku Oxford-Diffraction XCALIBUR E CCD diffractometer (Rigaku Oxford Diffraction, Sevenoaks, Kent, UK) equipped with graphite-monochromated MoKα radiation. The unit cell determination and data integration were carried out using the CrysAlis package of Oxford Diffraction [47]. The structures were solved by Intrinsic Phasing using Olex2 [48] software with the SHELXT [49] structure solution program and refined by full-matrix least-squares on F^2^ with SHELXL-2015 [50] using an anisotropic model for non-hydrogen atoms. All H atoms attached to carbon were introduced in idealized positions (d_CH_ = 0.96 Å) using the riding model. The molecular plots were obtained using the Olex2 program. Table 1 provides a summary of the crystallographic data together with refinement details for compounds. The geometric parameters are summarized in Table S1. The values of the geometrical parameters are in the expected ranges for such kinds of compounds. The supplementary crystallographic data can be obtained free of charge via www.ccdc.cam.ac.uk/conts/retrieving.html (accessed on 16 June 2021) (or from the Cambridge Crystallographic Data Centre, 12 Union Road, Cambridge CB2 1EZ, UK; fax: (+44)-1223-336-033; or deposit@ccdc.ca.ac.uk).

Hirshfeld was employed as implemented in CystalExplorer [51,52]. Hirshfeld surface maps highlight intermolecular interactions at the sum of d_e_ and d_i_, the distances from the external atoms to the surface or internal atoms to the Hirshfeld surface, respectively [52]. Distances shorter than the sum of the vdW radii are represented by red spots, close to the vdW radii in white spots and larger than vdW as blue surfaces. The fingerprint plots [52] show a qualitative description (see Supplementary Materials) of the relevant contacts in the crystal packing, by plotting d_i_ vs. d_e_, creating thus a “heatmap” of interactions.

### 3.1. Procedures for Synthesis of Acids ***1**–**3***

*N-(2-Fluorophenyl)glycine* (**1**) 40 mL (46 g; 0.41 mol) 2-fluoroaniline and 20 g (0.21 mol) monochloroacetic acid were refluxed in 300 mL water for 3 h. The reaction mixture was cooled in a water-ice bath and the precipitate was filtered by suction and then was washed with water on the filter. After drying the product was filtered. Brown crystals with mp 128–129 °C (lit.^45^ 127 °C) were obtained by recrystallization from benzene; Yield 60%. ^1^H NMR (300 MHz, DMSO) δ: 3.85 (s, 2H, CH_2_); 5.63 (bs, 1H, NH); 6.53–6.61 (m, 2H, H-4′, H-6′); 6.92–7.03 (m, 2H, H-3′, H-5′); ^13^C NMR (75 MHz, DMSO) δ: 44.2 (CH_2_); 112.1 (*J* = 3.7 Hz, C-6′); 114.4 (*J* = 18.0 Hz, C-3′); 116.1 (*J* = 6.9 Hz, C-4′); 124.7 (*J* = 3.1 Hz, C-5′); 136.3 (*J* = 11.6 Hz, C-1′); 151.0 (*J* = 237.0 Hz, C-2′); 172.5 (COOH).

*N-(4-Bromo-2-fluorophenyl)glycine* (**2**) A solution of 2.6 mL (8 g, 50 mmol) of bromine in 10 mL of glacial acetic acid was dropped under stirring to a suspension of 8.5 g (50 mmol) of *N*-(2-fluorophenyl)glycine in 25 mL of glacial acetic acid. Stirring was continued for 10 min. The reaction mixture was poured into water and the precipitate was filtered at vacuum. Light brown crystals with mp 138–143 °C were obtained by crystallization from benzene; Yield 78%. Anal. Calc. C_8_H_7_BrFNO_2_: C 38.74, H 2.84, N 5.65. Found: C 38.98, H 4.06, N 5.76. ^1^H NMR (300 MHz, DMSO) δ: 3.85 (s, 2H, CH_2_); 5.63 (bs, 1H, NH); 6.53–6.59 (m, 1H, H-3′); 7.11–7.14 (m, 1H, H-6′); 7.30 (dd, 1H, *J* = 11.5, 2.7 Hz, H-5′). ^13^C NMR (75 MHz, DMSO) δ: 44.0 (CH_2_); 105.4 (*J* = 9.2 Hz, C-4′); 113.5 (*J* = 4.6 Hz, C-6′); 117.4 (*J* = 21.7 Hz, C-3′); 127.4 (*J* = 3.3 Hz, C-5′); 136.0 (*J* = 11.0 Hz, C-1′); 150.6 (*J* = 242.0 Hz, C-2′); 172.1 (COOH).

*N-(4,6-Dibromo-2-fluorophenyl)glycine* (**3**) A solution of 4.4 mL (13.5 g, 80 mmol) of bromine in 10 mL of glacial acetic acid was dropped under stirring to a suspension of 6.8 g (40 mmol) of *N*-(2-fluorophenyl)glycine in 25 mL of glacial acetic acid. Stirring was continued for 30 min. The reaction mixture was poured into water and the precipitate was filtered under vacuum. Brown crystals with mp 148–150 °C were obtained by crystallization from benzene; Yield 90%. Anal. Calc. C_8_H_6_Br_2_FNO_2_: C 29.39, H 1.85, N 4.28. Found: C 29.68, H 1.95, N 4.51. ^1^H NMR (300 MHz, DMSO) δ: 4.03 (d, 2H, *J* = 4.7 Hz, CH_2_); 5.63 (bs, 1H, NH); 7.37 (dd, 1H, *J* = 13.0, 2.3 Hz, H-3′); 7.50 (dd, 1H, *J* = 2.3, 1.6 Hz, H-5′); ^13^C NMR (75 MHz, DMSO) δ: 46.5 (d, *J* = 9.8 Hz, CH_2_); 106.7 (d, *J* = 10.9 Hz, C-4′); 111.1 (d, *J* = 6.7 Hz, C-6′); 119.1 (d, *J* = 24.9 Hz, C-3′), 130.2 (d, *J* = 3.0 Hz, C-5′); 134.0 (d, *J* = 10.6 Hz, C-1′); 150.7 (d, *J* = 245.5 Hz, C-2′); 172.3 (d, *J* = 2.1 Hz, COOH).

### 3.2. Procedures for Synthesis of Sydnones ***4a**–**c***

To a solution of 2 g NaOH in 30 mL of water were added under stirring 20 mmol *N*-arylglycine **1**–**3** and 1.4 g (21 mmol) of NaNO_2_. In the cooled solution 10 mL of HCl were dropped under stirring, the temperature being maintained at 5–7 °C. The nitroso derivatives, separated as oils were extracted twice with CH_2_Cl_2_, and the organic layer was dried on CaCl_2_. The solvent was evaporated in vacuum on a water bath. The residue was treated with 30 mL of acetic anhydride and 2 mL of pyridine and evaporated under reduced pressure. The crude products were crystallized from a suitable solvent. 

*3-(2-Fluorophenyl)sydnone* (**4a**). Colorless crystals with mp 111–114 °C (Lit.^45^ 109 °C) were obtained by crystallization from ethanol; Yield 71%. ^1^H NMR (300 MHz, CDCl_3_) δ: 6.80 (d, 1H, *J* = 2.2 Hz, H-4); 7.37–7.44 (m, 2H, H-3′, H-6′); 7.62–7.71 (m, 1H, H-4′); 7.76–7.81 (m, 1H, H-5′). ^13^C NMR (75 MHz, CDCl_3_) δ: 97.1 (*J* ~ 0.7 Hz, C-4); 117.9 (*J* = 20.0 Hz, C-3′); 123.0 (*J* = 8.9 Hz, C-1′); 125.0 (*J* ~ 0.9 Hz, C-6′); 125.8 (*J* = 3.8 Hz, C-5′); 134.0 (*J* = 8.3 Hz, C-4′); 154.4 (*J* = 257.4 Hz, C-2′); 168.8 (CO).

*3-(4-Bromo-2-fluorophenyl)sydnone* (**4b**). Colorless crystals with mp 121–125 °C were obtained by crystallization from isopropanol; Yield 80%. Anal. Calc. C_8_H_4_BrFN_2_O_2_: C 37.09, H 1.56, N 10.81. Found: C 37.37, H 1.84, N 11.13. ^1^H NMR (300 MHz, CDCl_3_) δ: 6.81 (d, 1H, *J* = 2.2 Hz, H-4); 7.58–7.65 (m, 2H, H-3′, H-5′); 7.69–7.74 (m, 1H, H-6′). ^13^C NMR (75 MHz, CDCl_3_) δ: 97.0 (C-4); 121.6 (*J* = 22.0 Hz, C-3′, C-1′); 125.7 (C-6′); 127.3 (*J* = 9.1 Hz, C-4′); 129.1 (*J* = 3.8 Hz, C-5′); 153.9 (*J* = 262.0 Hz, C-2′); 168.4 (CO).

*3-(2,4-Dibromo-6-fluorophenyl)sydnone* (**4c**). Colorless crystals with mp 199–202 °C were obtained by crystallization from acetic acid; Yield 77%. Anal. Calc. C_8_H_3_Br_2_FN_2_O_2_: C 28.43, H 0.89, N 8.29. Found: C 28.72, H 1.27, N 8.58. ^1^H NMR (400 MHz, DMSO) δ: 6.73 (s, 1H, H-4); 8.22 (dd, 1H, *J* = 9.1, 1.9 Hz, H-3′); 8.27 (m, 1H, H-5′); ^13^C NMR (125 MHz, DMSO) δ: 99.4 (C-4); 120.5 (*J* = 22.3 Hz, C-3′); 121.2 (C-6′); 121.9 (*J* = 14.9 Hz, C-1′) 127.5 (*J* = 10.0 Hz, C-4′); 132.2 (*J* = 3.6 Hz, C-5′); 156.0 (*J* = 261.0 Hz, C-2′); 167.9 (CO).

### 3.3. Genereal Procedure for Synthesis of Pyrazoles ***5a**–**c***

A mixture of 5 mmol sydnone **4** and 0.9 g (6 mmol) of DMAD was refluxed 8 h in 20 mL toluene for **4a**,**b** and xylene for **4c**. After removal of the solvent in vacuo, the pyrazoles **5a**–**c** were crystallized from 2-propanol (**5a**) or ethanol (**5b** and **5c**).

*1-(2-Fluorophenyl)-3,4-dicarbomethoxypyrazole* (**5a**). Light brown crystals with mp 55–57 °C were obtained by crystallization from isopropanol; Yield 80%. Anal. Calc. C_13_H_11_FN_2_O_4_: C 56.12, H 3.98, N 10.07. Found: C 56.40, H 4.23, N 10.37. ^1^H NMR (300 MHz, CDCl_3_) δ: 3.87, 3.98 (2s, 6H, OCH_3_); 7.22–7.30 (m, 2H, H-3′, H-6′); 7.34–7.42 (m, 1H, H-4′); 7.89 (td, 1H, *J* = 7.9, 1.7 Hz, H-5′); 8.43 (d, 1H, *J* = 2.5 Hz, H-5). ^13^C NMR (75 MHz, CDCl_3_) δ: 52.2, 52.9 (2OCH_3_); 116.3 (C-4); 116.9 (*J* = 20.0 Hz, C-3′); 125.1 (C-6′); 125.2 (*J* = 3.6 Hz, C-5′); 129.7 (*J* = 9.4 Hz, C-1′); 129.8 (*J* = 8.0 Hz, C-4′); 135.7 (*J* = 10.0 Hz, C-5); 144.7 (C-3); 153.8 (*J* = 251.0 Hz, C-2′); 161.7, 162.0 (2COO).

*1-(4-Bromo-2-fluorophenyl)-3,4-dicarbomethoxypyrazole* (**5b**). Colorless crystals with mp 90–91 °C were obtained by crystallization from ethanol; Yield 71%. Anal. Calc. C_13_H_10_FBrN_2_O_4_: C 43.72, H 2.82, N 7.84. Found: C 43.97, H 3.11, N 8.09. ^1^H NMR (300 MHz, CDCl_3_) δ: 3.89, 4.00 (2s, 6H, OCH_3_); 7.45–7.49 (m, 2H, H-3′, H-5′); 7.80–7.85 (m, 1H, H-6′); 8.43 (d, 1H, *J* = 2.5 Hz, H-5). ^13^C NMR (75 MHz, CDCl_3_) δ: 52.2, 52.9 (OCH_3_); 116.6 (C-4); 120.6 (*J* = 22.0 Hz, C-3′); 122.0 (*J* = 8.8 Hz, C-4′); 126.0 (*J* = 0.7 Hz, C-6′); 126.2 (*J* = 9.4 Hz, C-1′); 128.7 (*J* = 3.3 Hz, C-5′); 136.5 (*J* = 10.0 Hz, C-5); 144.8 (C-3); 154.3 (*J* = 257.2 Hz, C-2′); 161.6, 161.9 (2COO).

*1-(2,4-Dibromo-6-fluorophenyl)-3,4-dicarbomethoxypyrazole* (**5c**). Colorless crystals with mp 151–154 °C were obtained by crystallization from ethanol; Yield 71%. Anal. Calc. C_13_H_9_Br_2_FN_2_O_4_: C 35.81, H 2.08, N 6.42. Found: C 36.11, H 2.34, N 6.71. ^1^H NMR (300 MHz, CDCl_3_) δ: 3.88, 3.97 (2s, 6H, OCH_3_); 7.43 (dd, 1H, *J* = 8.3, 1.9 Hz, H-3′); 7.71 (t, 1H, *J* = 1.9 Hz, H-5′); 8.07 (s, 1H, H-5). ^13^C NMR (75 MHz, CDCl_3_) δ: 52.1, *52*.8 (OCH_3_); 116.4 (C-4); 119.7 (*J* = 22.0 Hz, C-3′); 123.2 (C-6′); 125.0 (*J* = 10.1 Hz, C-4′); 126.7 (*J* = 14.8 Hz, C-1′); 131.7 (*J* = 3.6 Hz, C-5′); 137.1 (C-5); 145.1 (C-3); 157.9 (*J* = 262.2 Hz, C-2′); 161.4, 161.5 (2COO).

## 4. Conclusions

In conclusion, new polyhalogenated *N*-arylglycines, 3-arylsydnones and 1-arylpyrazoles having a fluorine atom on the *ortho* position of the phenyl ring were obtained and structurally characterized by ^1^H and ^13^C NMR spectroscopy. The NMR spectra were not trivial and present corresponding features of heteronuclear spin-spin coupling. The long range coupling between the H-4 or H-5 of the sydnone/pyrazole and the fluorine atom could test the presence of the hindered rotation between the phenyl and the sydnone/pyrazole in compound **3** having a bromine atom in position 6′. Halogen–halogen or halogen–π type contacts were identified either in phenylglycines or sydnones. In some cases, even the fluorine atom participates in a synergic mode to the halogen–halogen interactions. Pyrazoles are important benchmarks for the investigation of the halogen bonding, and we will continue to synthesize and investigate such molecules in order to bring some new information regarding its predictability. 

## Figures and Tables

**Table 1:** ^13^C NMR assignments and the multiplicity according to ^19^F-^13^C spin–spin coupling for the compounds **1**–**3**, **4a**–**c** and **5a**–**c**.

No.	C-3	C-4	C-5	C-1′	C-2′	C-3′	C-4′	C-5′	C-6′
	Chemical Shift (ppm), ^19^F-^13^C Coupling Constant *J* (Hz)								
**1** [45]	-	-	-	136.3 *J* = 11.6	151.0 *J* = 237.7	114.4 *J* = 18.0	116.1 *J* = 6.9	124.7 *J* = 3.2	112.1 *J* = 3.8
**2**	-	-	-	136.0 *J* = 11.5	150.6 *J* = 242.0	117.4 *J* = 21.8	105.4 *J* = 9.2	127.4 *J* = 3.7	113.5 *J* = 4.6
**3**	-	-	-	134.0 *J* = 10.6	150.7 *J* = 245.5	119.1 *J* = 24.9	106.7 *J* = 10.9	130.2 *J* = 3.0	111.1 *J* = 6.7
**4a** [45]	-	97.1 *J* ~ 0.7	-	123.0 *J* = 8.9	154.4 *J* = 257.4	117.9 *J* = 20.0	134.0 *J* = 8.3	125.8 *J* = 3.8	125.0 *J* ~ 0.9
**4b**	-	97.0 *J* ~ 0.7	-	121.4 *J* = 9.0	153.9 *J* = 262.0	121.6 *J* = 22.0	127.3 *J* = 9.1	129.1 *J* = 3.8	125.7 Small *J*
**4c**	-	99.4 No *J*	-	121.9 *J* = 14.9	156.0 *J* = 261.0	120.5 *J* = 22.3	127.5 *J* = 10.0	132.2 *J* = 3.6	121.2 Small *J*
**5a**	144.7	116.3	135.7 *J* = 10.0	129.7 *J* = 9.4	153.8 *J* = 251.0	116.9 *J* = 20.0	129.8 *J* = 8.0	125.2 *J* = 3.6	125.1 Small *J*
**5b**	144.8	116.5	136.5 *J* = 10.0	126.2 *J* = 9.4	154.3 *J* = 257.2	120.6 *J* = 22.0	122.0 *J* = 8.8	128.7 *J* = 3.4	126.0 *J* ~ 0.7
**5c**	145.1	116.4	137.1 No *J*	126.7 *J* = 14.8	157.9 *J* = 262.2	119.7 *J* = 22.0	125.0 *J* = 10.1	131.7 *J* = 3.6	123.2 Small *J*

**Table 2:** The structures of the compounds **3** and **4a**–**c** and X-ray diffraction crystal parameters for each compound.

Parameter	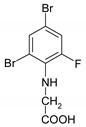 3	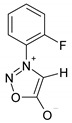 4a	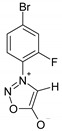 4b	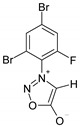 4c
Empirical formula	C_8_H_6_Br_2_FNO_2_	C_8_H_5_FN_2_O_2_	C_8_H_4_BrFN_2_O_2_	C_8_H_3_Br_2_FN_2_O_2_
*Fw*	326.96	180.14	259.04	337.94
space group	*P*-1	*P*2_1_/*c*	*I*2/*a*	*P*2_1_/*n*
*a* [Å]	8.8727(6)	6.7072(5)	13.8035(9)	10.3170(8)
*b* [Å]	10.4079(7)	12.6000(11)	8.6487(4)	9.1652(5)
*c* [Å]	12.7466(11)	9.3015(6)	14.9402(7)	10.5526(8)
*α* [°]	107.218(7)	90	90	90
*β* [°]	91.685(6)	102.085(7)	95.109(5)	95.928(6)
*γ* [°]	114.901(7)	90	90	90
*V* [Å^3^]	1003.57(14)	768.66(10)	1776.51(17)	992.49(12)
*Z*	4	4	8	4
*r*_calcd_ [g cm^−3^]	2.164	1.557	1.937	2.262
Crystal size [mm]	0.30 × 0.20 × 0.20	0.30 × 0.10 × 0.10	0.30 × 0.20 × 0.20	0.30 × 0.20 × 0.20
*T* [K]	293	293	293	293
*μ* [mm^−1^]	8.064	0.131	4.616	8.161
2Θ range [°]	4.588 to 58.638	5.524 to 50.038	5.448 to 50.05	5.258 to 52.722
Reflections collected	11,043	5284	3760	9203
Independent reflections	4731[*R*_int_ = 0.0491]	1346[*R*_int_ = 0.0405]	1559[*R*_int_ = 0.0552]	2027[*R*_int_ = 0.0543]
Data/restraints/parameters	4731/0/255	1346/0/118	1559/0/127	2027/0/136
*R*_1_^a^	0.0580	0.0451	0.0334	0.0455
*wR*_2_^b^	0.1037	0.1150	0.0392	0.0670
GOF ^c^	0.992	1.098	1.021	1.076
Largest diff. peak/hole [e Å^−3^]	0.52/−0.48	0.17/−0.26	0.32/−0.49	0.49/−0.43
CCDC No.	2080828	2080829	2080830	2080831

^a^*R*_1_ = Σ||*F*_o_| − |*F*_c_||/Σ|*F*_o_|. ^b^ *wR*_2_ = {Σ[*w*(*F*_o_^2^ − *F*_c_^2^)^2^]/Σ[*w*(*F*_o_^2^)^2^]}^1/2^. ^c^ GOF = {Σ[*w*(*F*_o_^2^ − *F*_c_^2^)^2^]/(*n* − *p*)}^1/2^, where *n* is the number of reflections and *p* is the total number of parameters refined.

## Data Availability

Not applicable.

## Supplementary Materials

The following are available online. Figure S1: Partial view of 3D network in the crystal structure of compounds **3** (a), and **4b** (b). Interlayer centroid-tocentroid distances are showing in dashed-orange lines, Figure S2: Partial view of the crystal structure for compounds **4a** (a), and **4c** (b) showing the parallel packing of 2D double layers, Table S1: Deviations (Å) of the atoms from mean least-squares plane for molecule **3**, Table S2: Deviations (Å) of the atoms from mean least-squares plane for molecule **4a**, **4b** and **4c**.

## Sample Availability

Samples of the compounds are available from the authors.
