# Supplementary material for: Synthesis of 1-(2-Fluorophenyl)pyrazoles by 1,3-Dipolar Cycloaddition of the Corresponding Sydnones
Source: Molecules. 2021 Jun 17;26(12):3693. doi: 10.3390/molecules26123693 (PMC8235238; doi:10.3390/molecules26123693)

Supporting Information

Synthesis of 1-(2-fluorophenyl)pyrazoles by 1,3-dipolar cycloaddition of the corresponding sydnones

Denisa Dumitrescu[1], Sergiu Shova[2,3], Constantin Draghici[4], Marcel Mirel Popa[4*], and Florea Dumitrascu [4*]

1 Ovidius University Constanta, Faculty of Pharmacy, Str. Cpt. Av. Al. Serbanescu, Campus Corp C, Constanta 900470, Romania
2 Ningbo University of Technology, No.55-155 Cui Bai Road, Ningbo City, Zhejiang, 315016, China
3 "Petru Poni" Institute for Macromolecular Chemistry, Romanian Academy, Department of inorganic polymers, Aleea Grigore Ghica Voda, 41A, 700487 Iasi, Romania
4 Center of Organic Chemistry ''C. D. Nenitzescu'', Roumanian Academy, Spl Independentei 202B, 060023 Bucharest, Romania

* **Correspondence: Correspondence: M.M.P. mirelupb@gmail.com; F.D. fdumitra@yahoo.com;**

1. X-Ray diffraction analysis

Table 1S. Deviations (Å) of the atoms from mean least-squares plane for molecule **3**.

atom	Part A	Part B
Br1*	-0.085	0.026
Br2*	0.061	0.184
C1*	-0.031	0.-0.103
C2*	0.001	-0.013
C3*	0.027	0.065
C4*	0.041	0.072
C5*	0.017	-0.008
C6*	-0.014	-0.099
C7*	-0.043	0.087
C8*	0.032	0.070
N1*	-0.065	-0.238
O1*	0.037	0.371
O2*	0.098	-0.186
RMS deviation of fitted atoms	0.053	0.160

*indicates atom used to define plane.

Table 2S.. Deviations (Å) of the atoms from mean least-squares plane for molecule **4a**, **4b** and **4c**.

atom	**4a**	**4b**	**4c**
Br1*	-	0.181	0.348
Br2*	-	-	-0.239
C1*	0.026	-0.001	0.147
C2*	0.240	0.245	0.161
C3*	0.276	0.292	0.049
C4*	0.100	0.105	-0.069
C5*	-0.112	-0.147	-0.068
C6*	-0.140	-0.203	0.030
C7*	0.435	0.613	-0.729
C8*	0.176	0.290	-0.180
N1*	-0.553	-0.801	1.381
O1*	-0.430	-0.605	1.157
O2*	0.341	0.576	-0.557
RMS deviation	0.294	0.405	0.626

of fitted atoms			

*indicates atom used to define plane

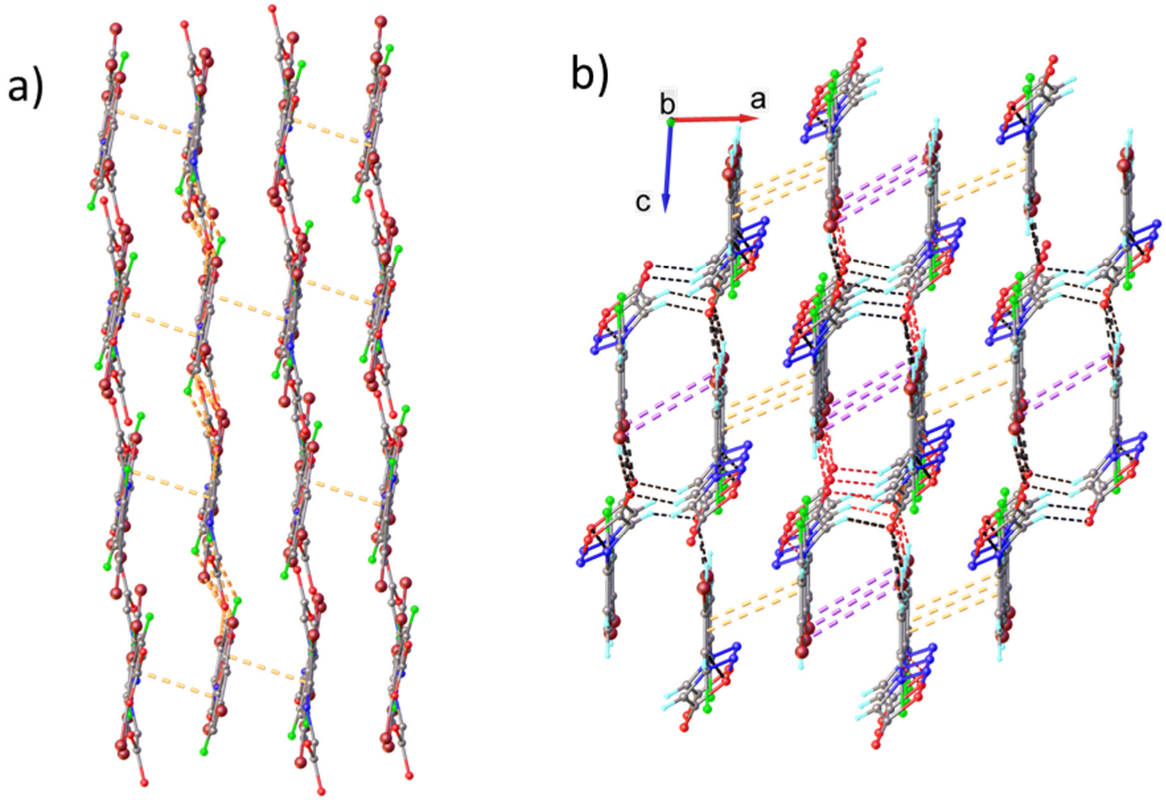

Figure 1S. Partial view of 3D network in the crystal structure of compounds 3 (a), and 4b (b). Interlayer centroid-tocentroid distances are showing in dashed-orange lines.

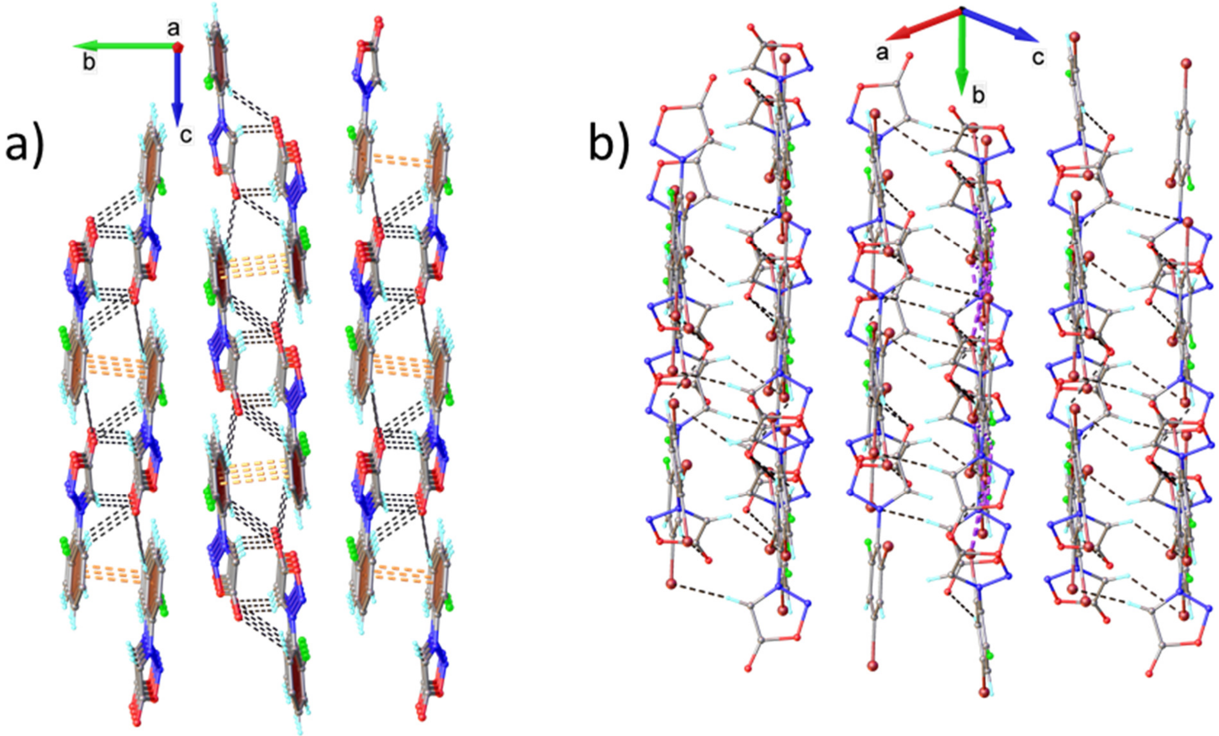

Figure 2S. Partial view of the crystal structure for compounds 4a (a), and 4c (b) showing the parallel packing of 2D double layers.

Table 3S Bond distances (Å) and angles (°).
Compound 3.

Bond	Molecule A	Molecule B
Br1-C2	1.892(5)	1.889(5)
Br2-C4	1.888(5)	1.889(6)
F1-C6	1.363(6)	1.366(6)
O1-C8	1.312(6)	1.324(6)
O2-C8	1.213(6)	1.198(6)
N1-C1	1.361(6)	1.370(6)
N1-C7	1.411(6)	1.434(6)
C1-C2	1.412(7)	1.402(7)
C1-C6	1.392(7)	1.381(8)
C2-C3	1.370(7)	1.382(6)
C3-C4	1.359(7)	1.361(7)
C4-C5	1.381(8)	1.391(8)
C5-C6	1.359(7)	1.372(7)
C7-C8	1.497(7)	1.508(7)
**Angle**	**Molecule A**	**Molecule B**
C1-N1-C7	127.5(4)	124.4(5)
N1-C1-C2	121.6(5)	121.1(5)
N1-C1-C6	125.3(5)	125.1(5)
C6-C1-C2	113.1(5)	113.7(5)
C1-C2-Br1	117.7(4)	118.7(4)
C3-C2-Br1	119.0(4)	117.8(4)
C3-C2-C1	123.2(5)	123.6(5)
C4-C3-C2	120.1(6)	118.9(5)
C3-C4-Br2	120.7(5)	120.2(5)
C3-C4-C5	119.8(5)	120.9(5)
C5-C4-Br2	119.5(5)	118.9(5)
C6-C5-C4	118.8(5)	117.5(6)
F1-C6-C1	117.9(5)	118.5(5)
C5-C6-F1	117.1(5)	116.2(6)
C5-C6-C1	125.0(6)	125.3(6)
N1-C7-C8	110.1(4)	108.2(5)
O1-C8-C7	112.3(5)	111.3(5)
O2-C8-O1	124.4(5)	124.8(6)

O2-C8-C7	123.3(5)	123.8(5)

Compound 4a.

F1-C6	1.354(3)
O1-N1	1.379(2)
O1-C8	1.414(3)
O2-C8	1.220(3)
N1-N2	1.317(2)
N2-C1	1.444(3)
N2-C7	1.335(3)
C1-C2	1.379(3)
C1-C6	1.368(3)
C2-C3	1.388(3)
C3-C4	1.381(3)
C4-C5	1.380(3)
C5-C6	1.382(3)

N1-O1-C8	111.13(17)
N2-N1-O1	103.24(18)
N1-N2-C1	116.12(19)
N1-N2-C7	115.23(19)
C7-N2-C1	128.6(2)
C2-C1-N2	119.7(2)
C6-C1-N2	120.6(2)
C6-C1-C2	119.7(2)
C1-C2-C3	119.6(2)
C4-C3-C2	119.9(2)
C5-C4-C3	120.7(2)
C4-C5-C6	118.4(2)
F1-C6-C1	119.8(2)
F1-C6-C5	118.5(2)

C1-C6-C5	121.7(2)
N2-C7-C8	106.8(2)
O2-C8-O1	119.5(2)
O2-C8-C7	136.9(3)
C7-C8-O1	103.5(2)

Compound 4b.

Br1-C4	1.895(3)
F1-C6	1.352(3)
O1-N1	1.377(3)
O1-C8	1.418(3)
O2-C8	1.207(3)
N1-N2	1.322(3)
N2-C1	1.437(3)
N2-C7	1.336(3)
C1-C2	1.372(3)
C1-C6	1.374(3)
C2-C3	1.386(3)
C3-C4	1.375(3)
C4-C5	1.366(3)
C5-C6	1.372(3)
C7-C8	1.406(3)

N1-O1-C8	112.1(2)
N2-N1-O1	102.8(2)
N1-N2-C1	117.0(2)
N1-N2-C7	115.5(2)
C7-N2-C1	127.5(2)
C2-C1-N2	119.8(2)
C2-C1-C6	119.9(3)
C6-C1-N2	120.2(3)

**C1-C2-C3**	**119.5(3)**
**C4-C3-C2**	**119.1(3)**
**C3-C4-Br1**	**119.2(2)**
**C5-C4-Br1**	**118.8(2)**
**C5-C4-C3**	**121.9(3)**
**C4-C5-C6**	**118.1(3)**
**F1-C6-C1**	**119.4(2)**
**F1-C6-C5**	**119.2(2)**
**C5-C6-C1**	**121.4(3)**
**N2-C7-C8**	**106.9(3)**
**O2-C8-O1**	**120.1(3)**
**O2-C8-C7**	**137.2(3)**
**C7-C8-O1**	**102.8(3)**

Compound 4c

**Br1-C2**	**1.888(4)**
**Br2-C4**	**1.895(4)**
**F1-C6**	**1.343(4)**
**O2-C8**	**1.208(5)**
**N2-C1**	**1.440(5)**
**N2-C7**	**1.313(5)**
**N2-N1**	**1.322(6)**
**C1-C2**	**1.376(5)**
**C1-C6**	**1.379(5)**
**C2-C3**	**1.380(5)**
**C3-C4**	**1.367(5)**
**C4-C5**	**1.380(5)**
**C5-C6**	**1.374(5)**
**C7-C8**	**1.389(5)**
**C8-O1**	**1.394(9)**

**O1-N1**	**1.387(13)**

**C7-N2-C1**	**127.9(4)**
**C7-N2-N1**	**114.2(8)**
**N1-N2-C1**	**117.5(8)**
**C2-C1-N2**	**122.0(3)**
**C2-C1-C6**	**118.7(4)**
**C6-C1-N2**	**119.3(3)**
**C1-C2-Br1**	**120.3(3)**
**C1-C2-C3**	**120.6(4)**
**C3-C2-Br1**	**119.1(3)**
**C4-C3-C2**	**118.4(4)**
**C3-C4-Br2**	**118.7(3)**
**C3-C4-C5**	**123.1(4)**
**C5-C4-Br2**	**118.2(3)**
**C6-C5-C4**	**116.6(4)**
**F1-C6-C1**	**118.3(3)**
**F1-C6-C5**	**119.2(4)**
**C5-C6-C1**	**122.5(4)**
**N2-C7-C8**	**107.6(4)**
**O2-C8-C7**	**138.0(5)**
**O2-C8-O1**	**117.9(8)**
**C7-C8-O1**	**104.0(8)**
**N1-O1-C8**	**110.3(12)**
**N2-N1-O1**	**103.3(13)**

2. Hiershfeld analysis

Table 1. Fingerprint plots mapping the most important interactions in compounds **3** and **4a-c**

Compound 3 – independent molecule A

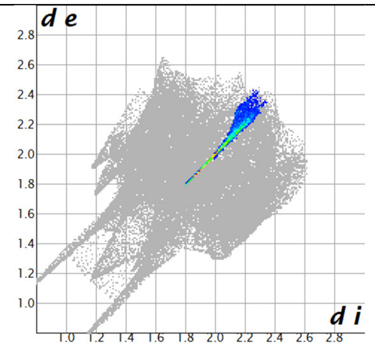

Br···Br - 6.8%

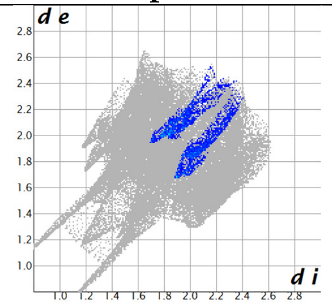

Br···C - 4.3%

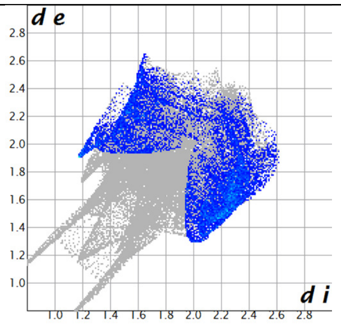

Br···H - 18%

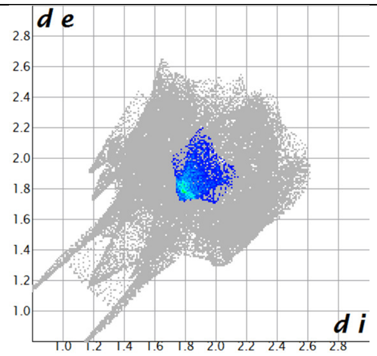

C···C - 6.3%

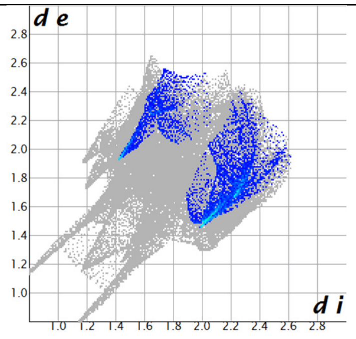

F···Br - 9.3%

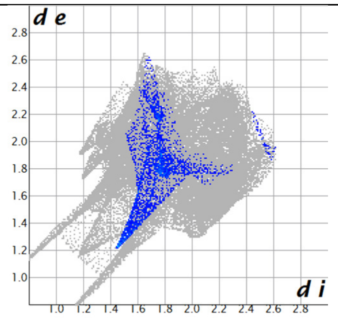

F···H - 4.8%

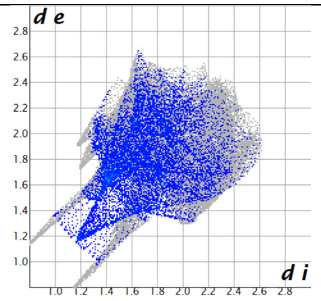

H···H - 16.4%

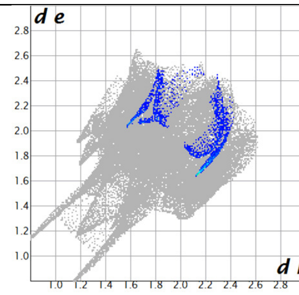

O···Br - 3.4&3.7%
Bifurcated bond

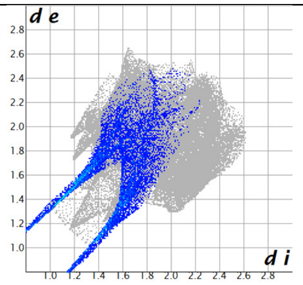

O···H - 14.2%

Compound 3 – independent molecule B

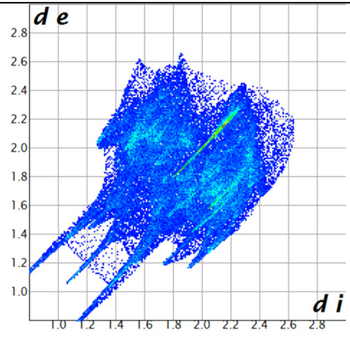

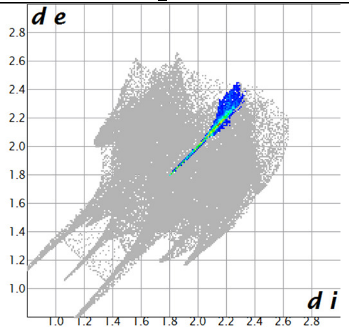

Br···Br - 6.5%

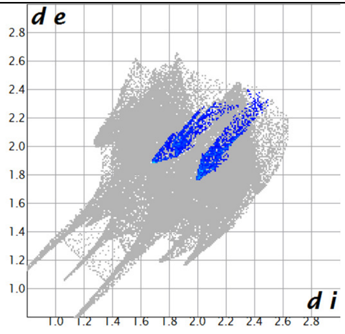

Br···C - 3.6%

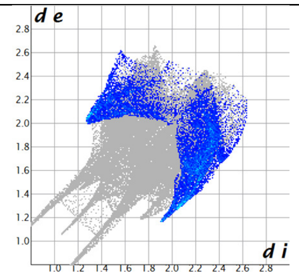	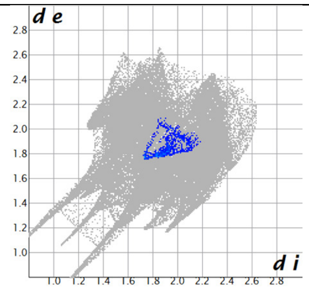	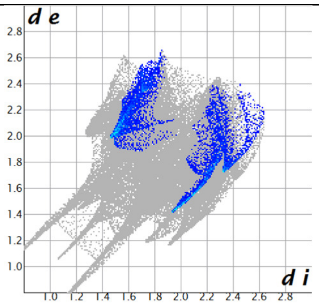
Br···H - 9.9%	C···C - 1.4%	F···Br - 9.1%
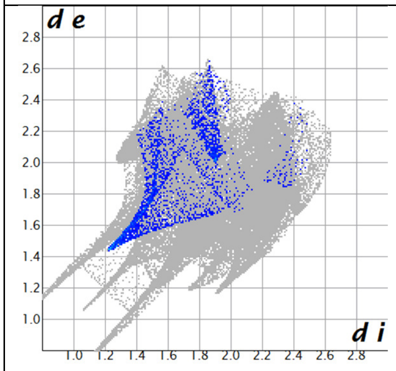	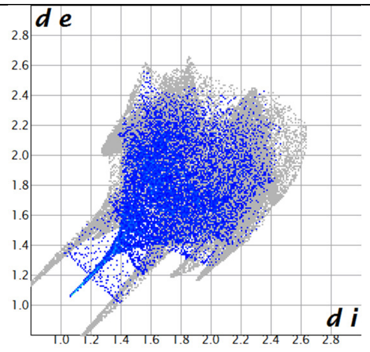	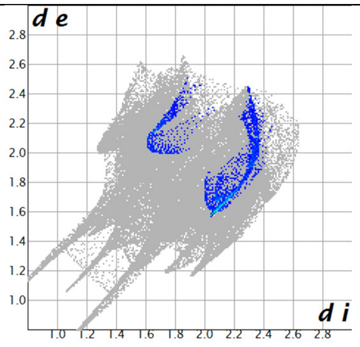
F···H - 4.1%	H···H - 18.2%	O···Br - 4.2%
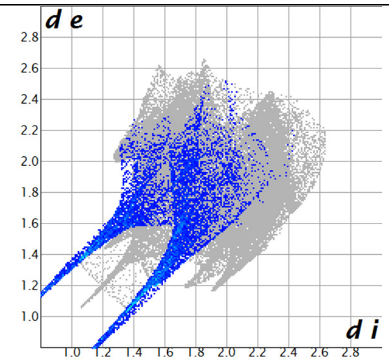		
O···H - 15%		
**Compound 4a**		
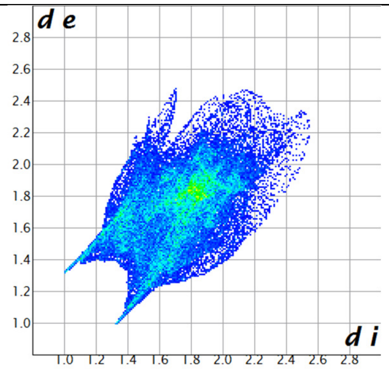	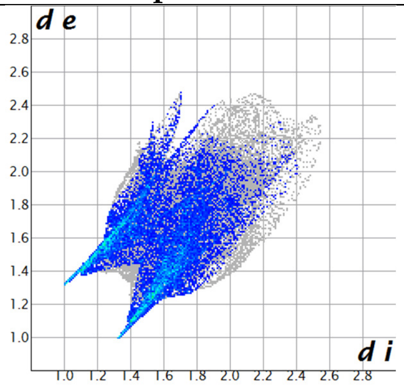	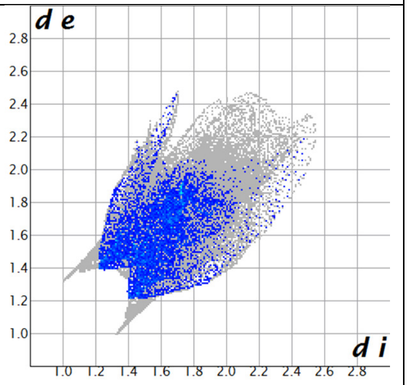
	O···H - 27.8%	F···H - 13.5%

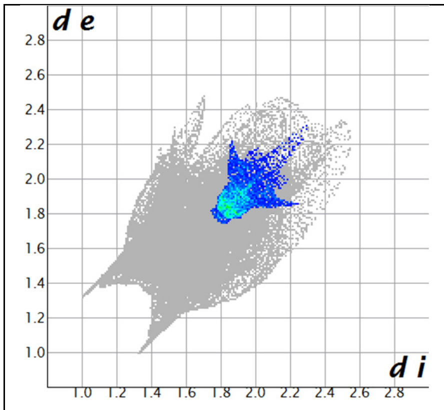	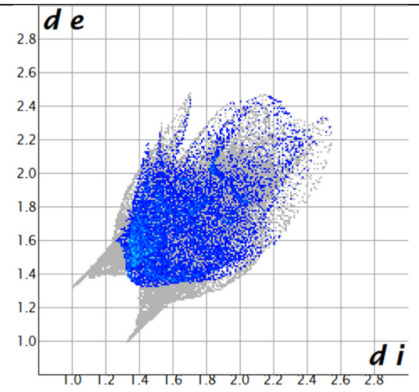	
C···C - 10%	H···H - 18.2%	
**Compound 4b**		
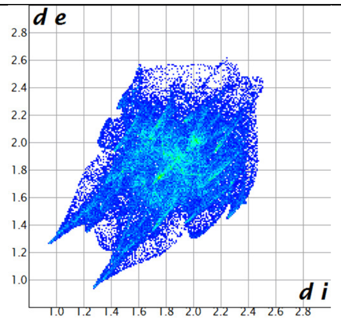	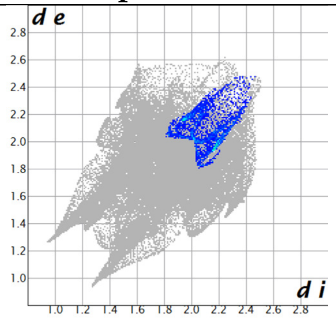	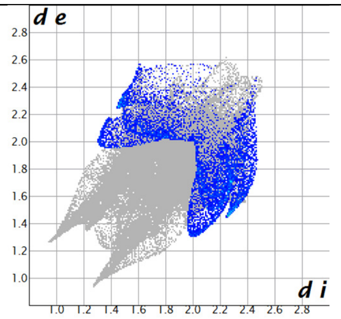
	Br···C - 4.7% Br···syd	Br···H - 11.7%
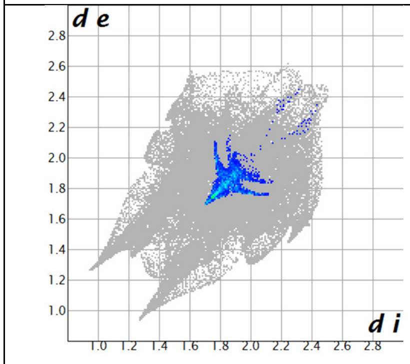	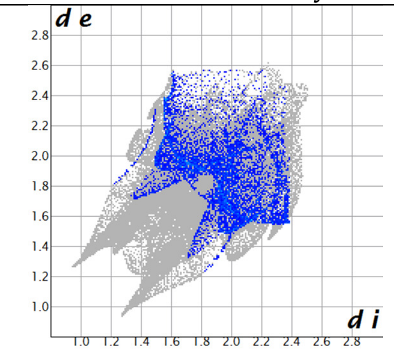	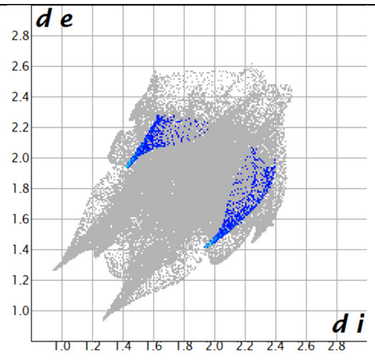
C···C - 4.9%	C···H - 11.9%	F···Br 2.5% at limit vdW
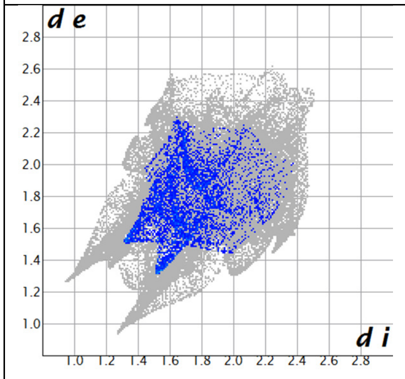	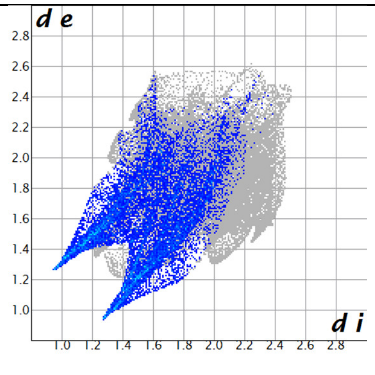	
F···H 8.3%	O···H 22.2%	
**Compound 4c**		

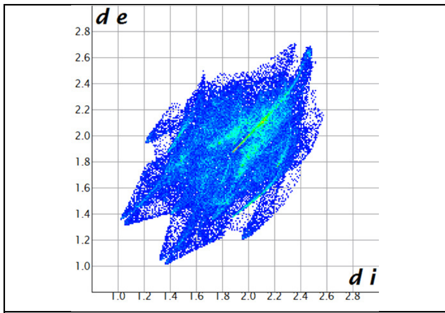	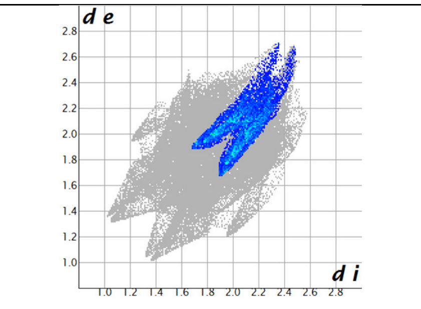	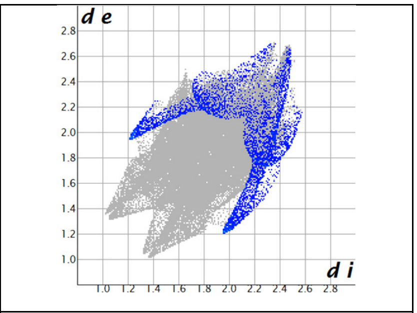
	Br···C - 17.2%	Br···H - 6.3%
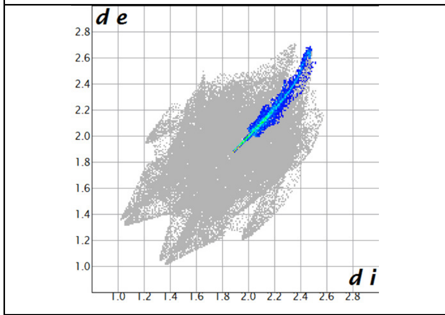	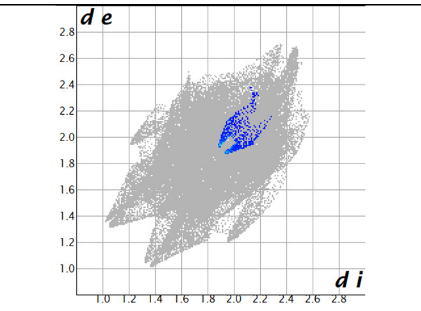	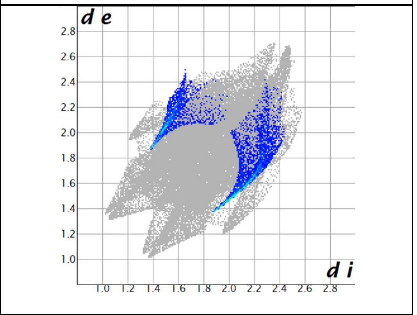
Br···Br - 7.1%	C···C - 1.4%	F···Br - 8%
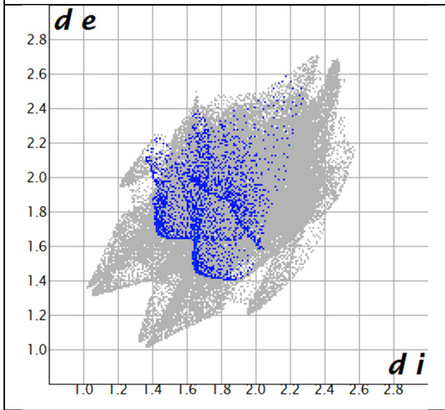	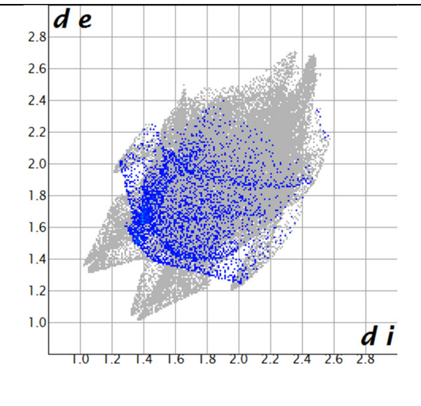	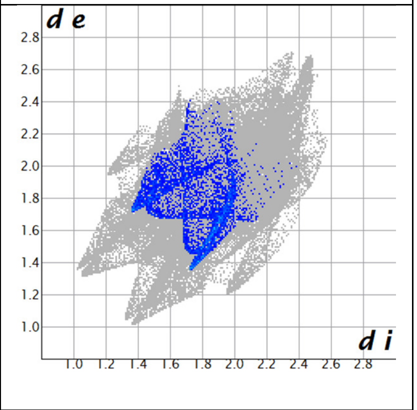
F···H - 3.6%	H···H - 5.2%	N···H - 7.1%
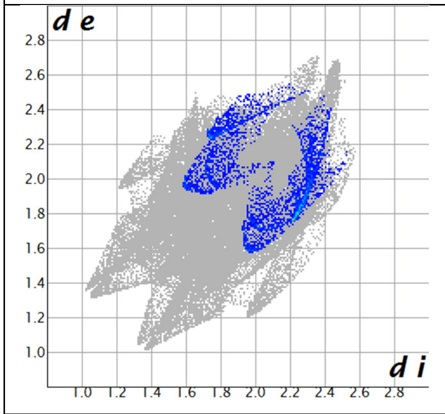	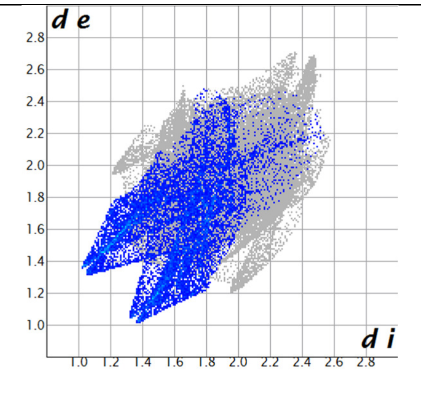	
O···Br - 5.1%	O···H - 18.9%	

3. NMR spectra

Compound 1

^{1}H-NMR spectrum of 1 recorded at 300 MHz (inset: aromatic protons)

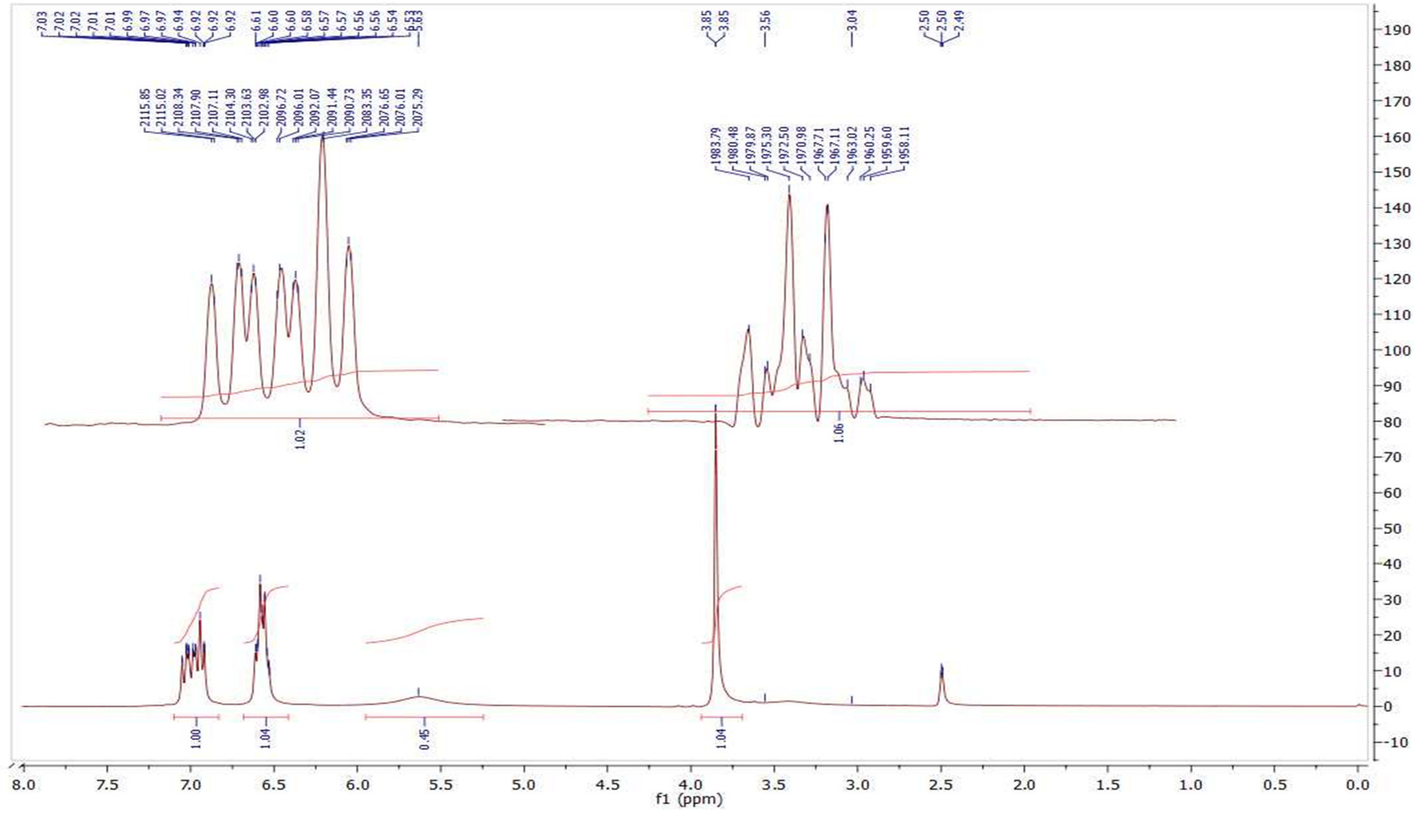

^{13}C-NMR spectrum of 1 recorded at 75 MHz (inset: aromatic C atoms)

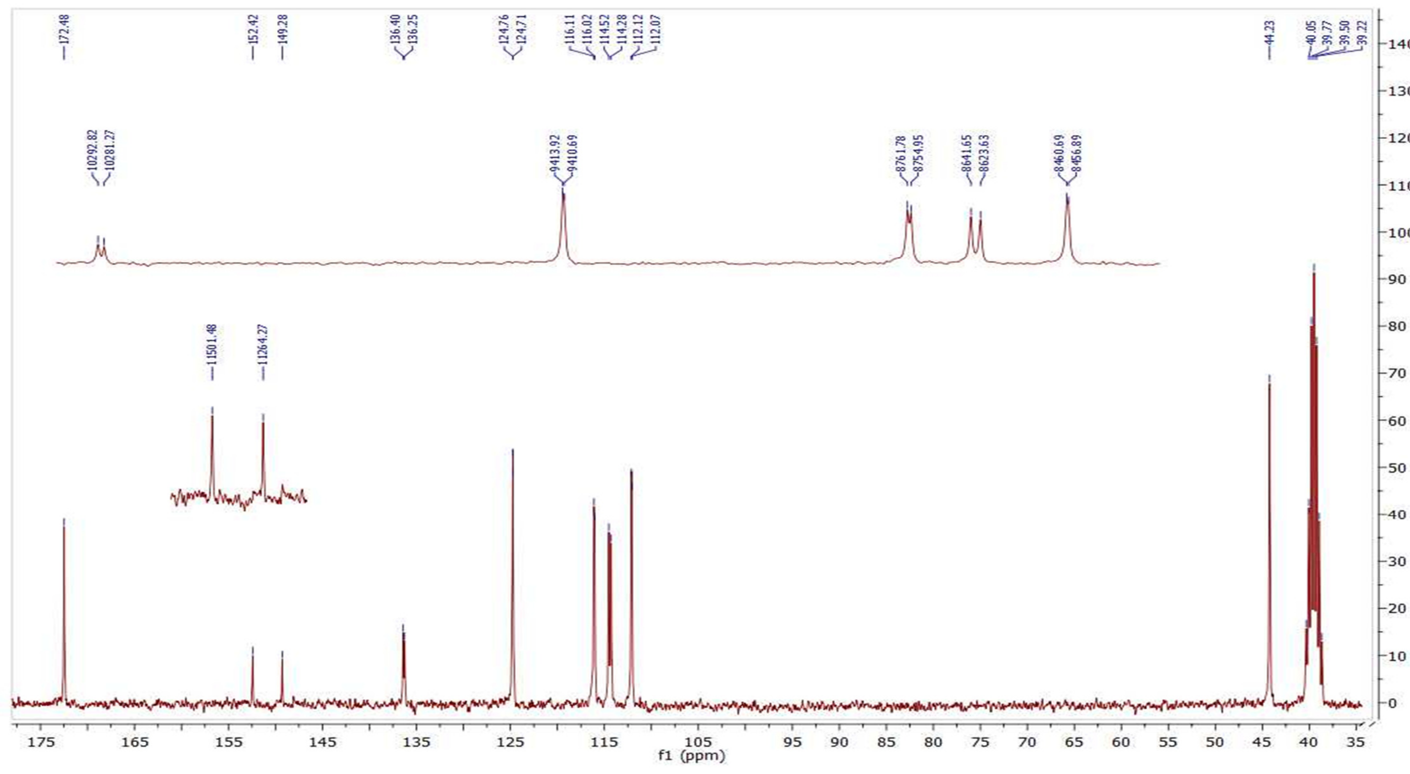

^{1}H-^{13}C Heteronuclear Correlation spectrum (HETCOR) of 1 presenting the ^{1}H-^{13}C signals for the aromatic region of the spectra

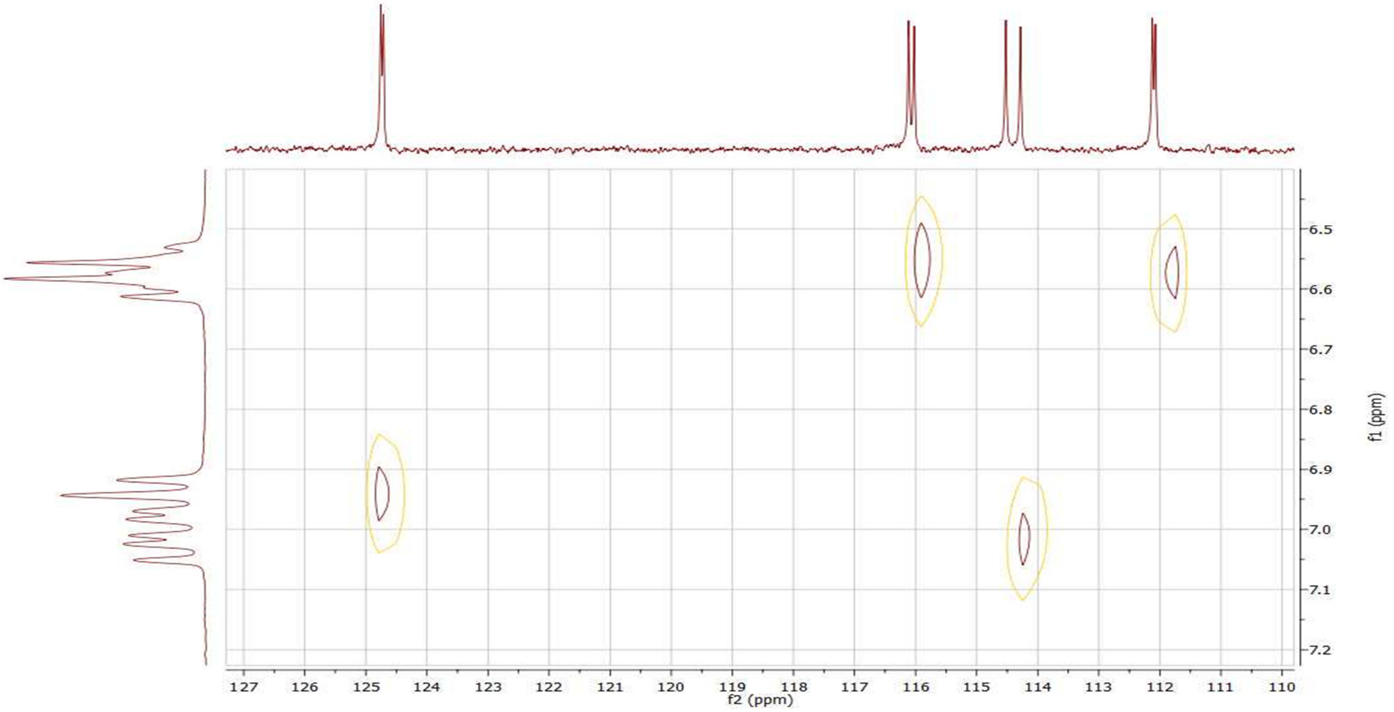

Compound 2

^{1}H-NMR spectrum of 2 recorded at 300 MHz

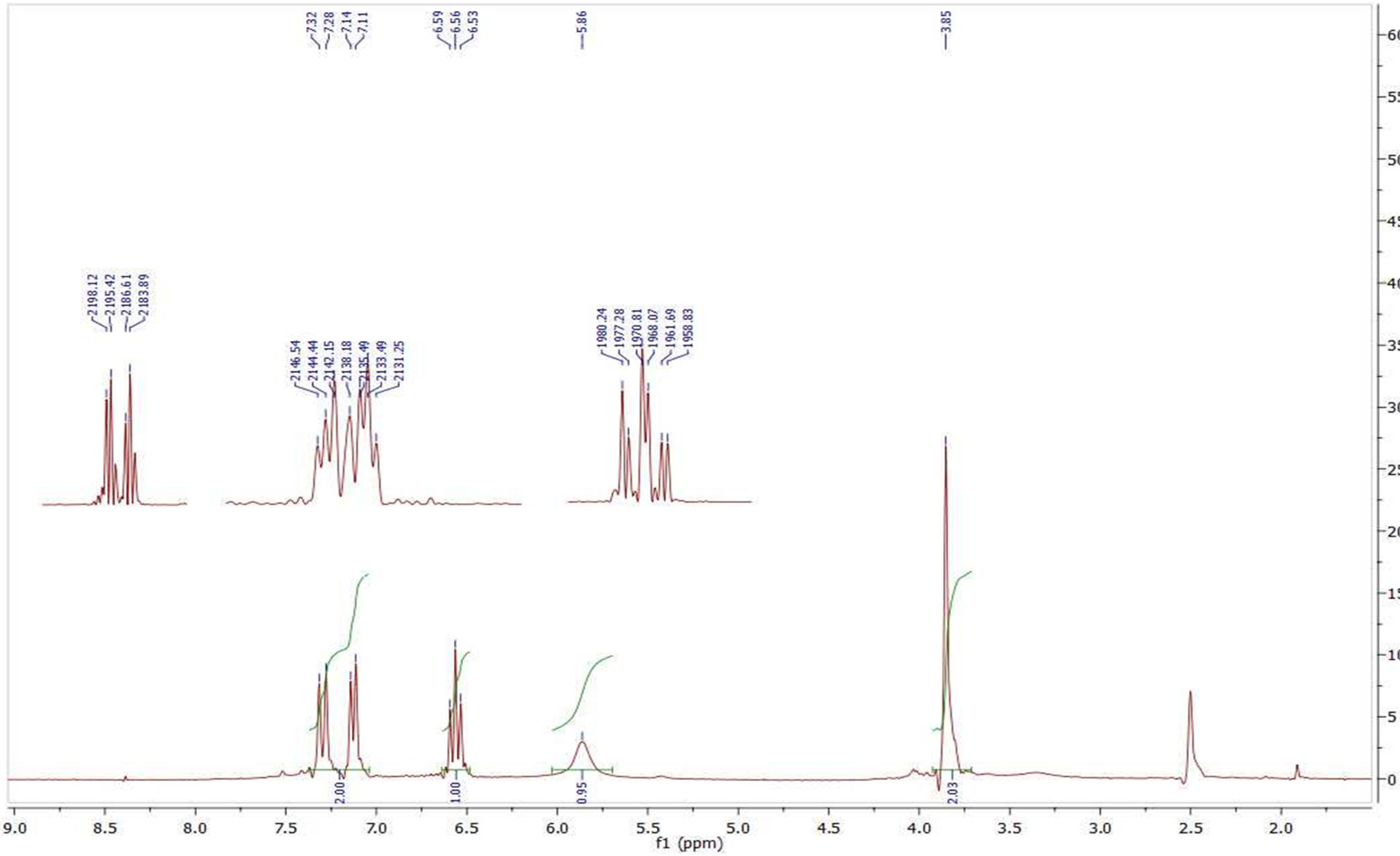

^{13}C-NMR spectrum of 2 recorded at 75 MHz

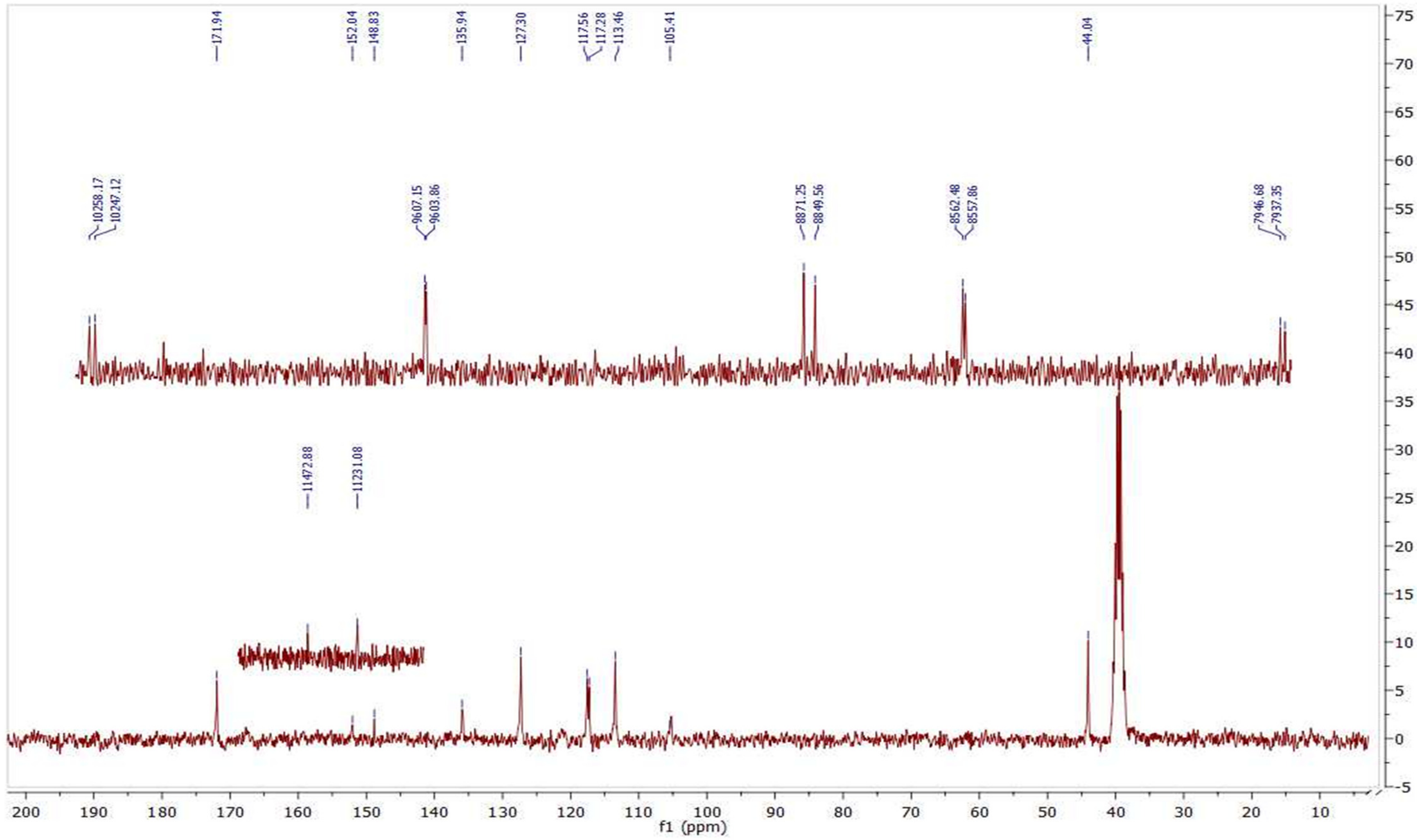

Compound 3

^{1}H-NMR spectrum of 3 recorded at 300 MHz

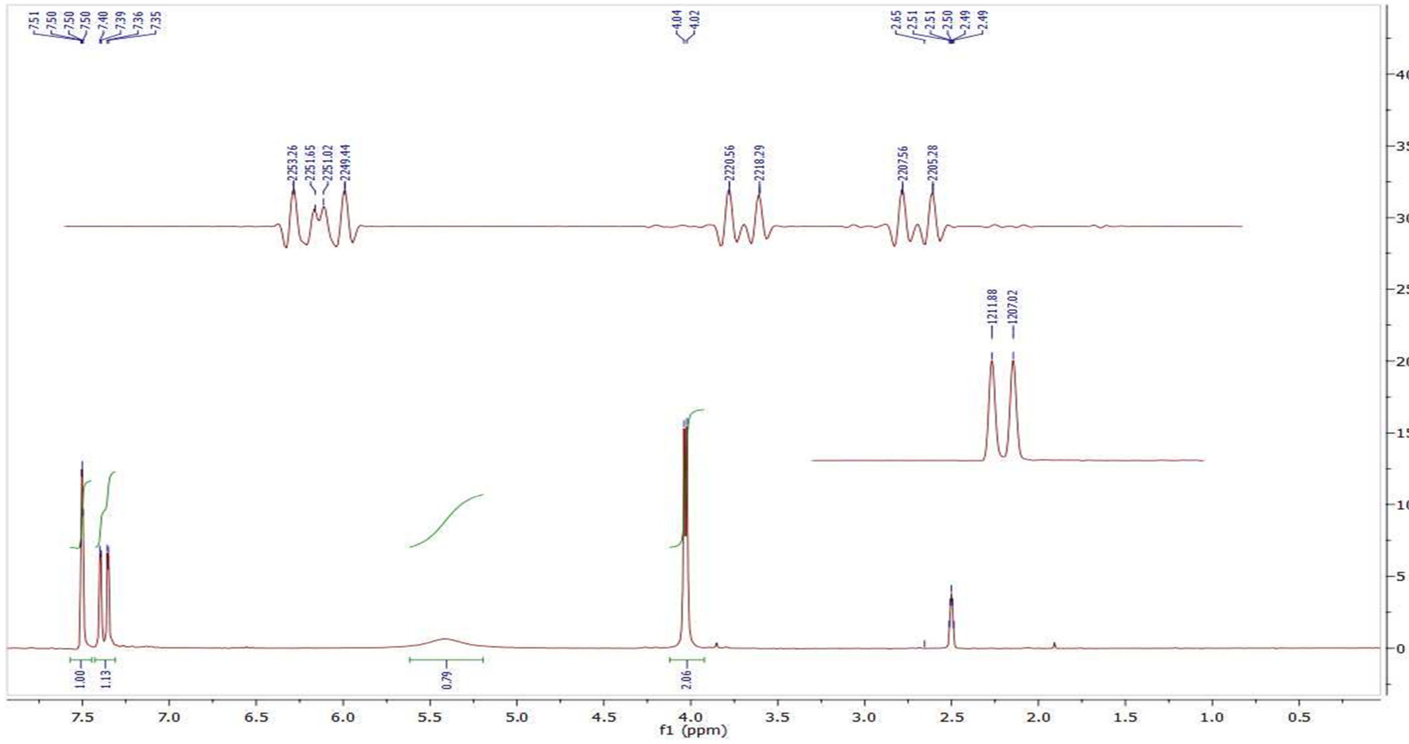

^{13}C-NMR spectrum of 3 recorded at 75 MHz

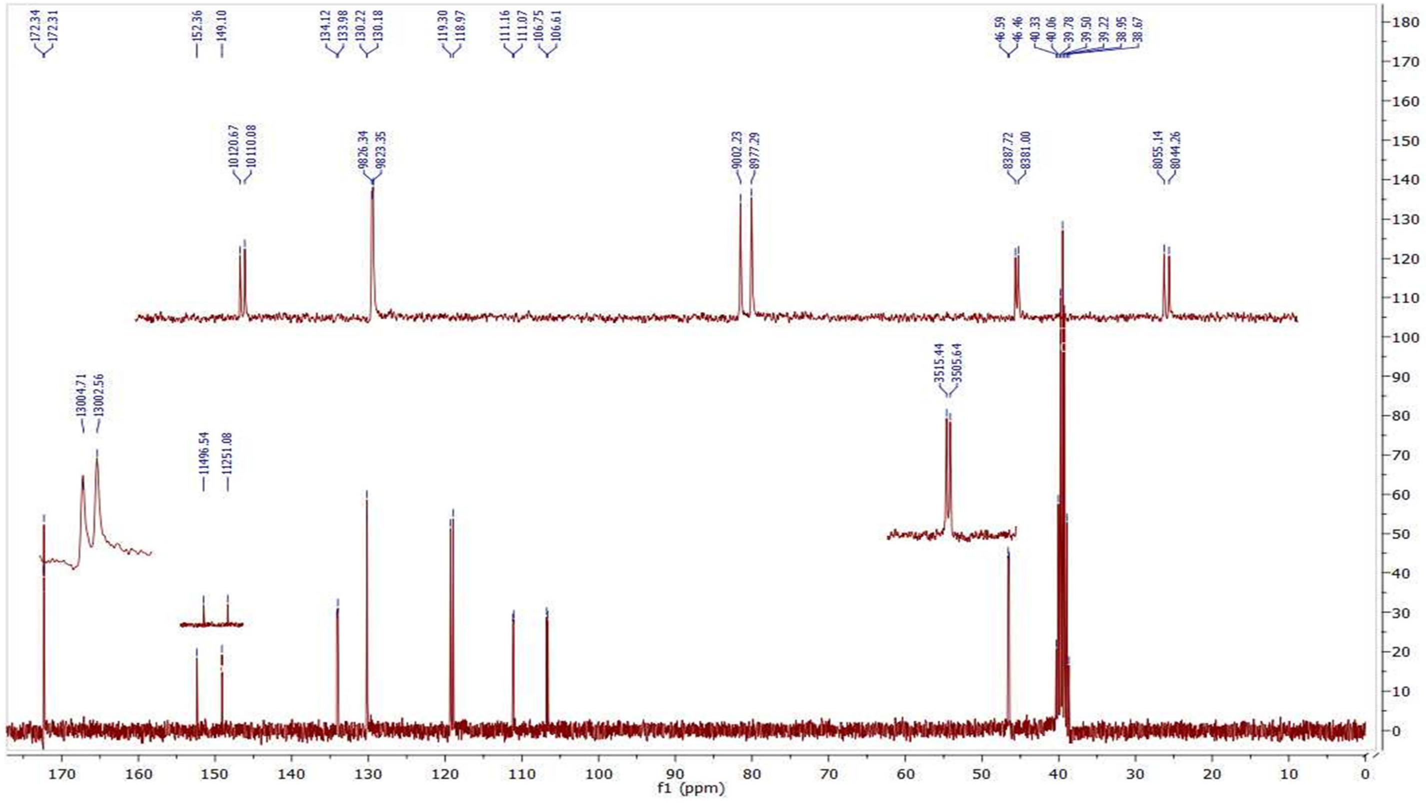

Compound 4a

^{1}H-NMR spectrum of 4a recorded at 300 MHz

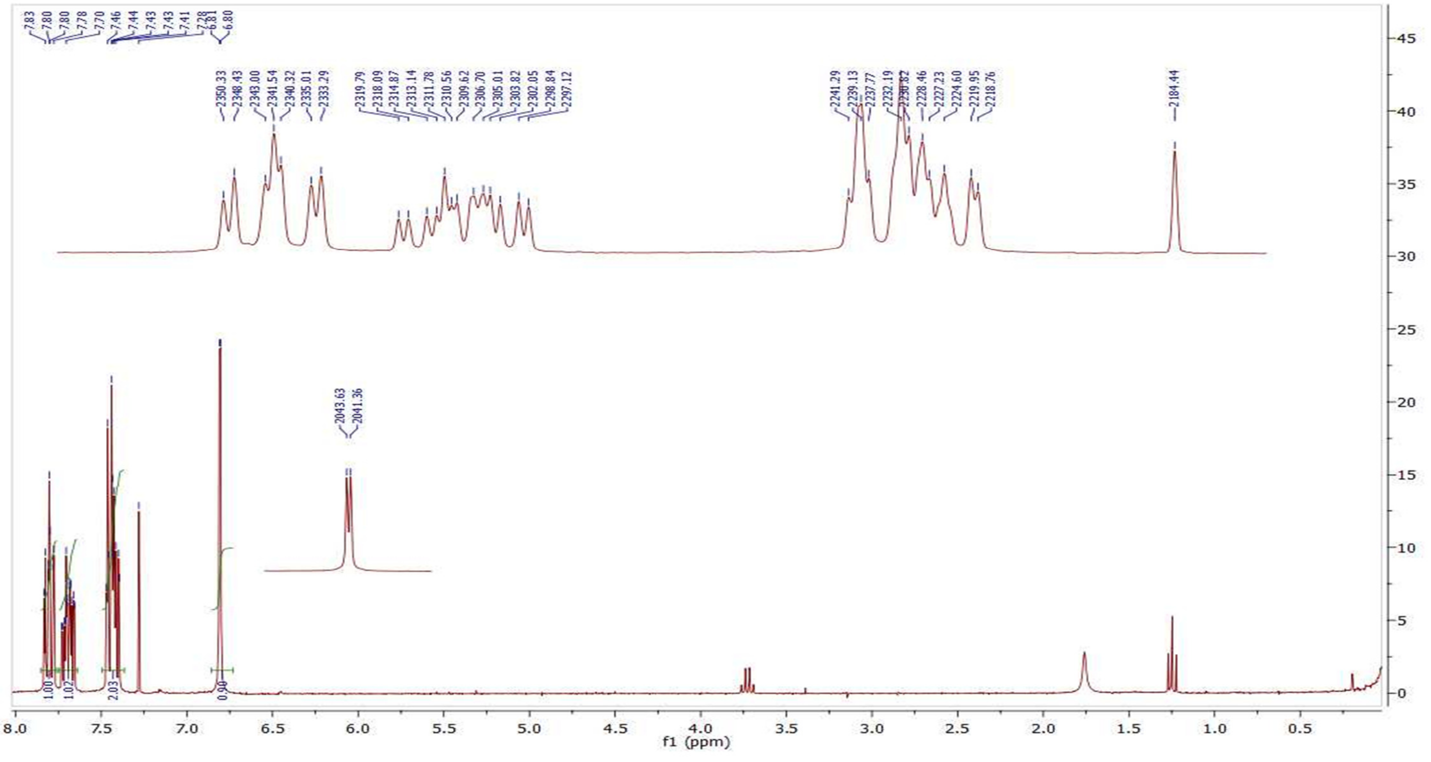

^{13}C-NMR spectrum of 4a recorded at 75 MHz

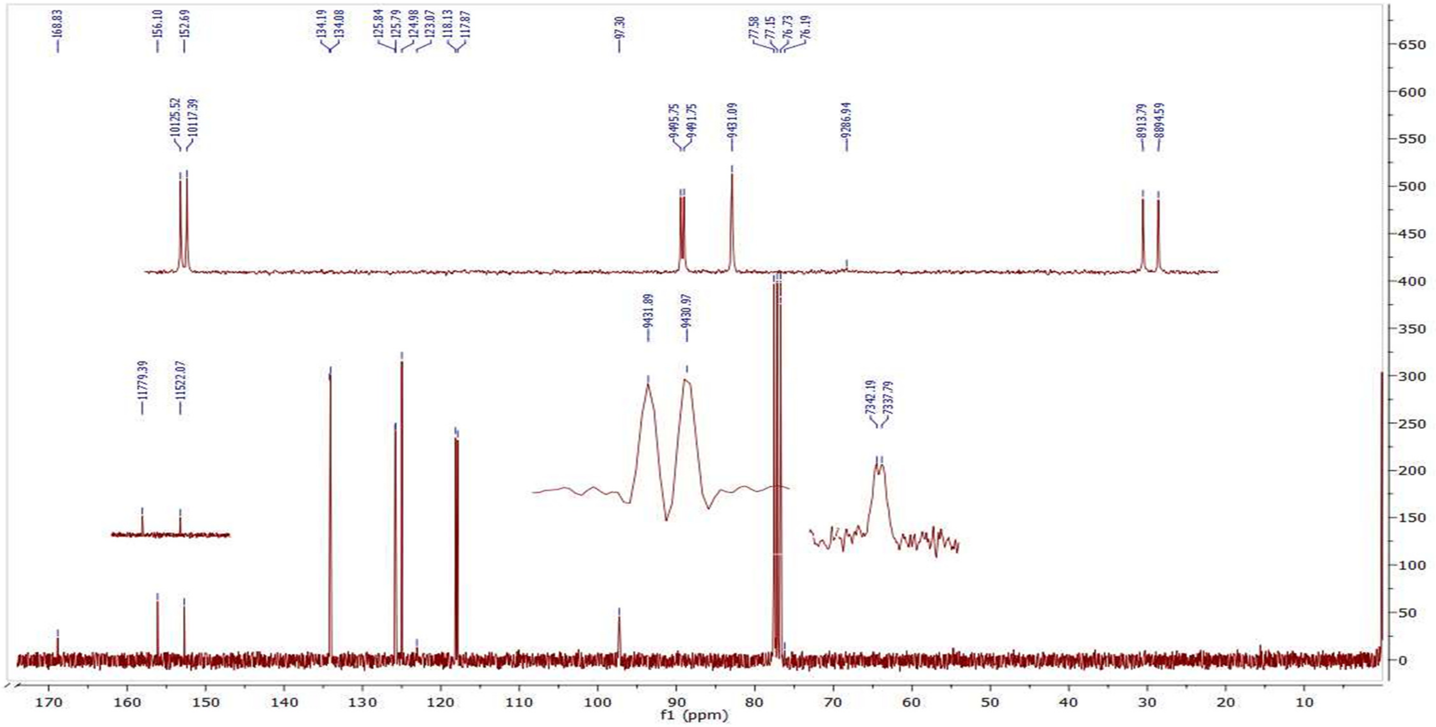

^{13}C-NMR spectrum of 4a recorded at 75 MHz (added small quantity of $Cr(acac)_3$ as NMR relaxation agent)

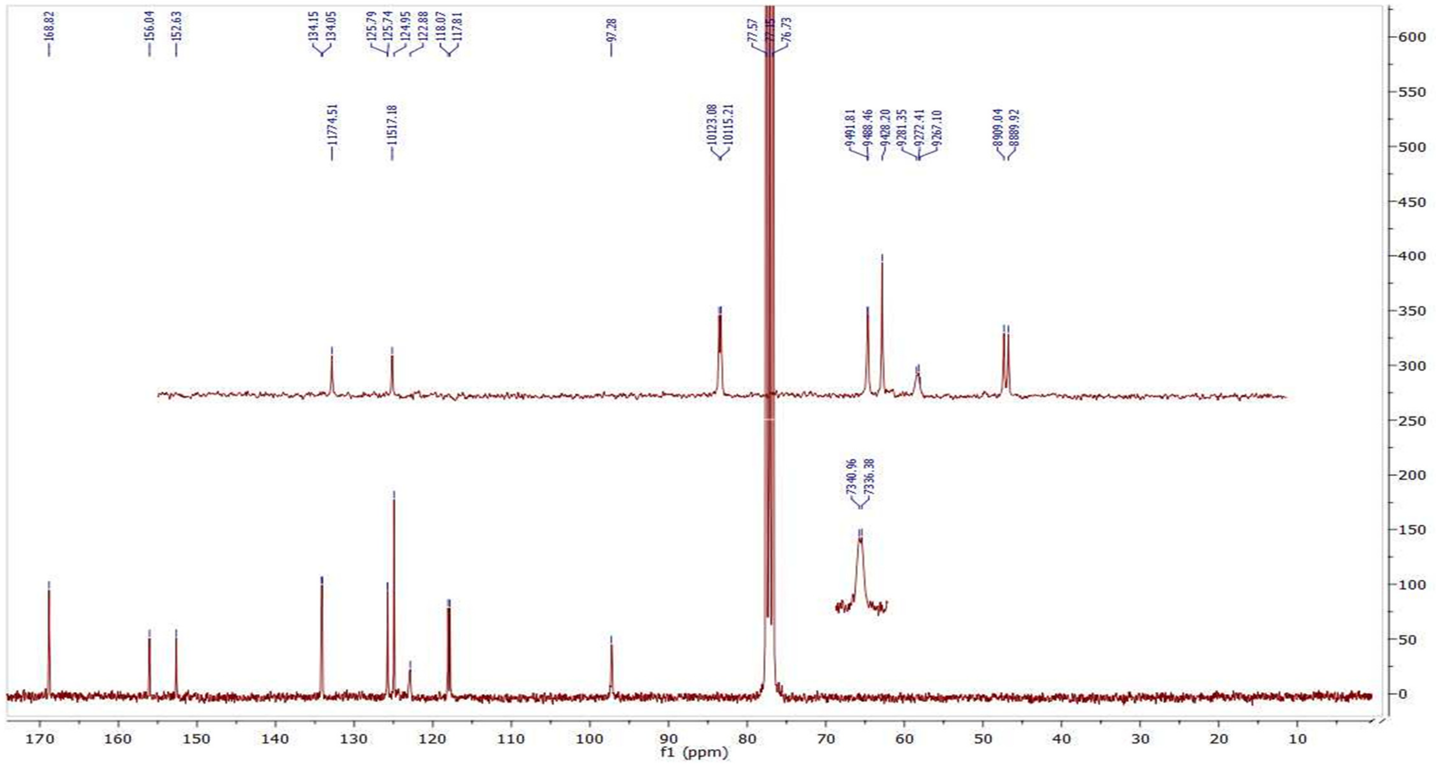

Compound 4b

^{1}H-NMR spectrum of 4b recorded at 300 MHz

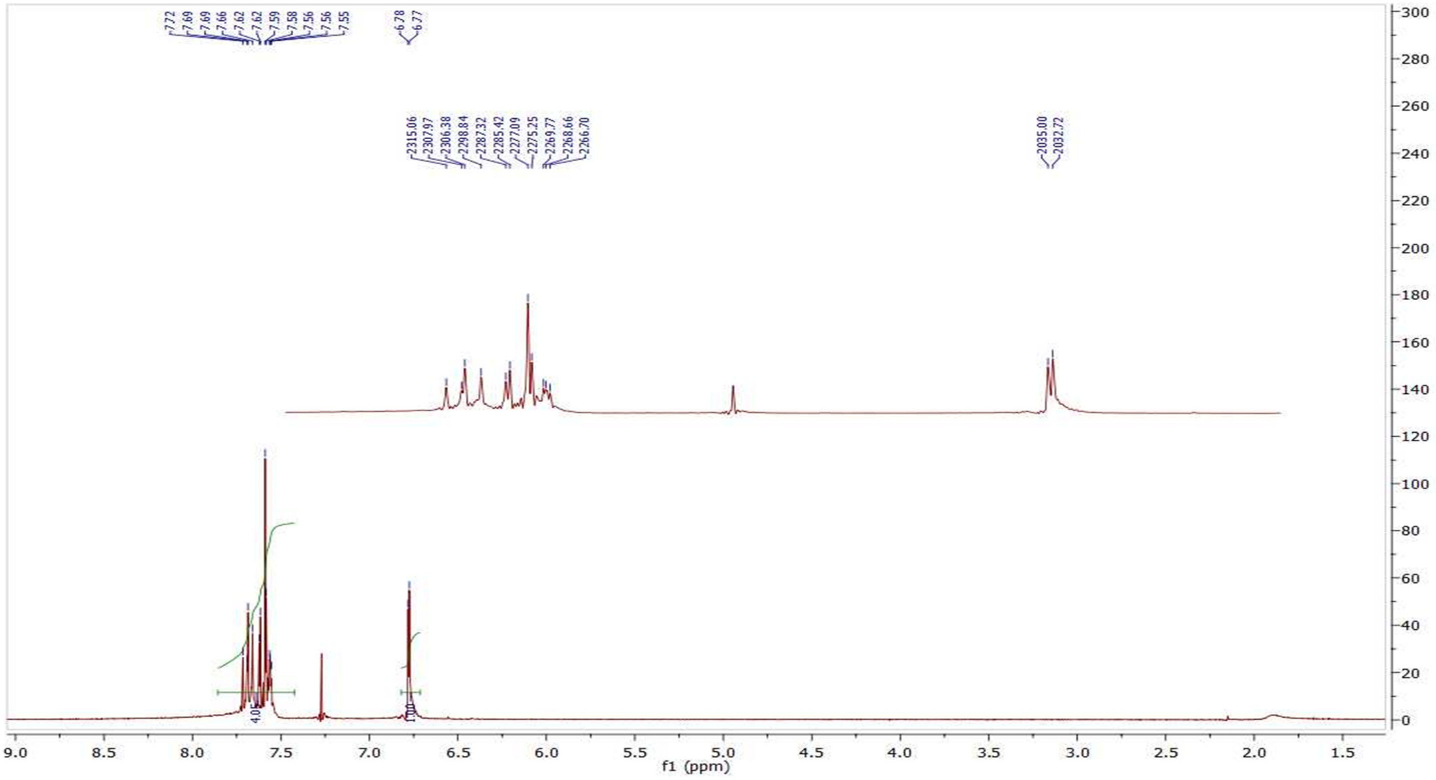

^{13}C-NMR spectrum of 4b recorded at 75 MHz

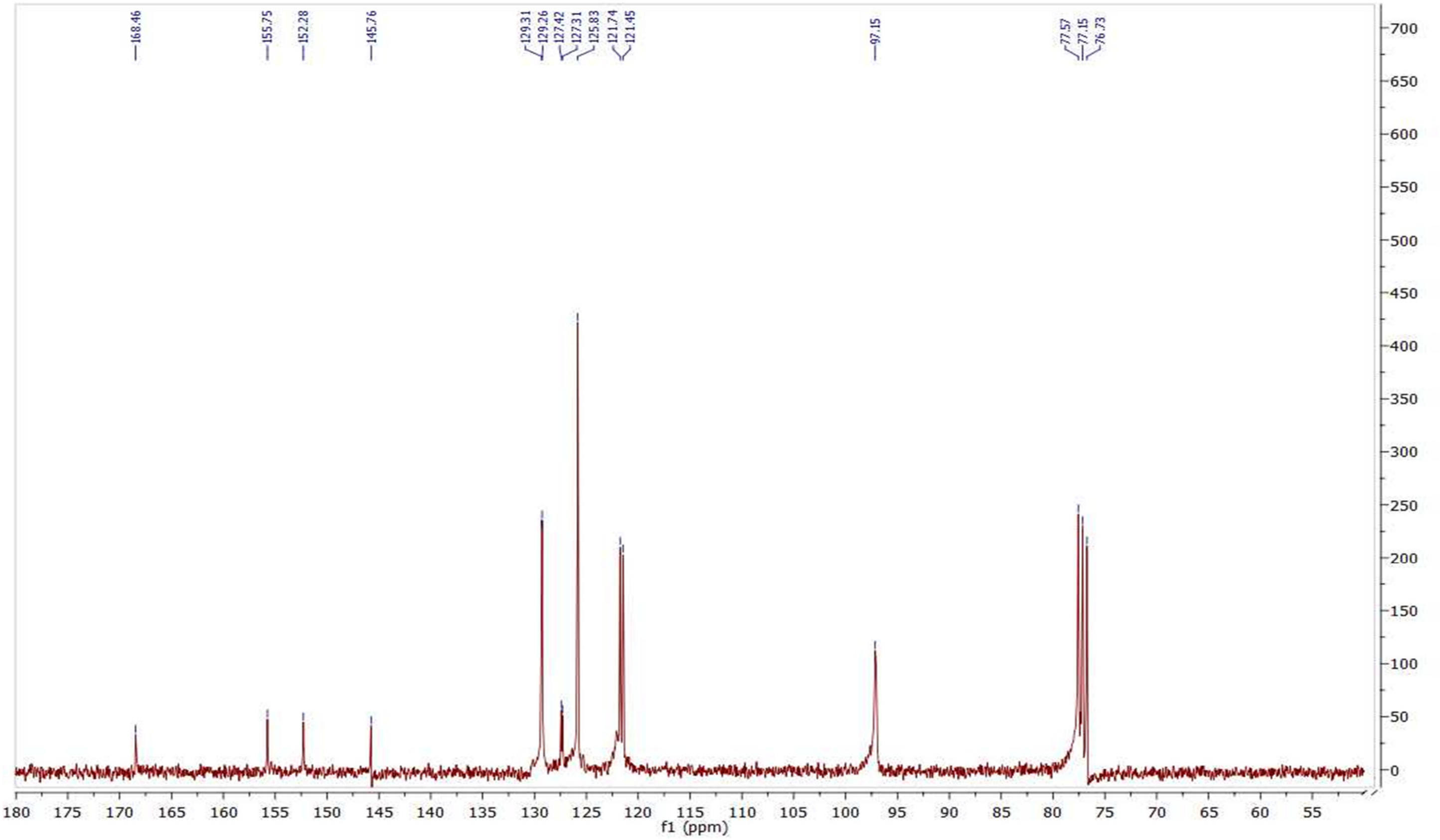

Compound 4c

^{1}H-NMR spectrum of 4c recorded at 400 MHz (DMSO)

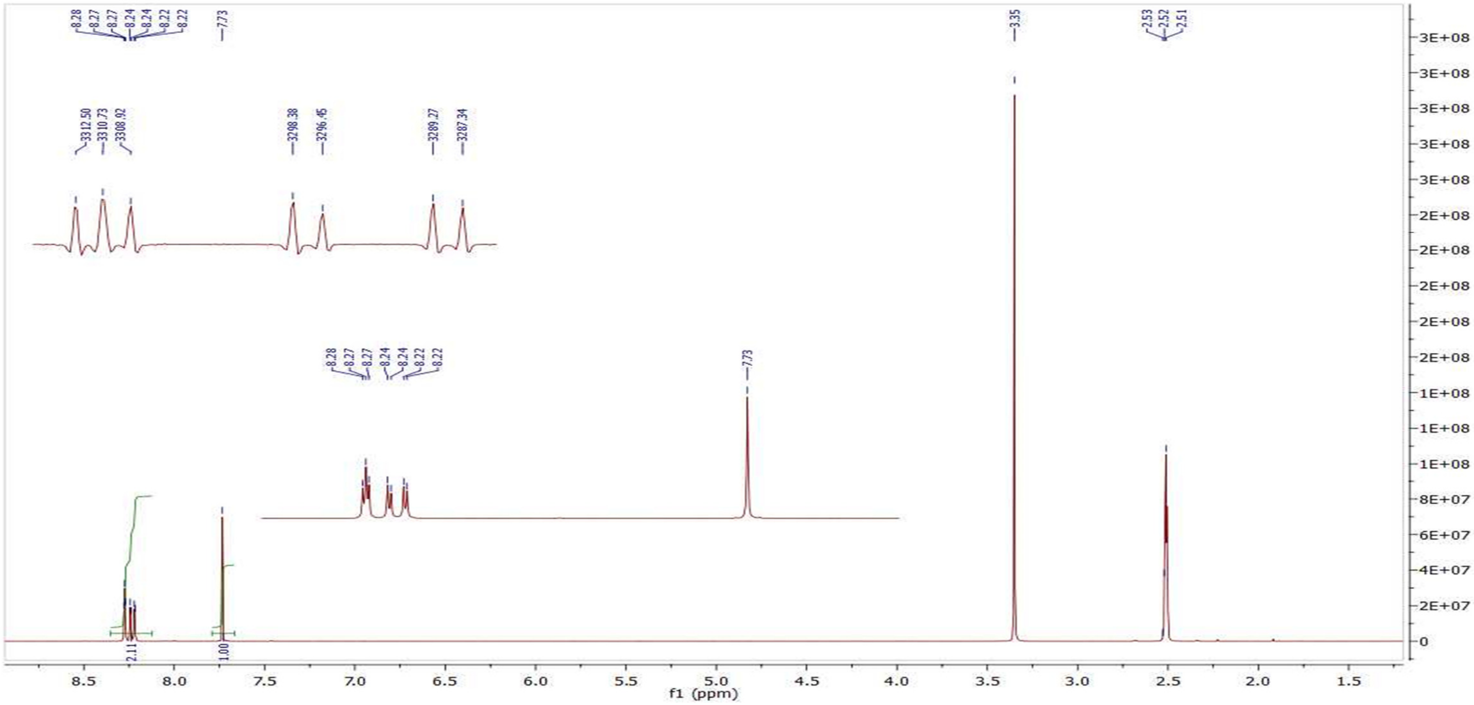

^{13}C-NMR spectrum of 4c recorded at 125 MHz (DMSO)

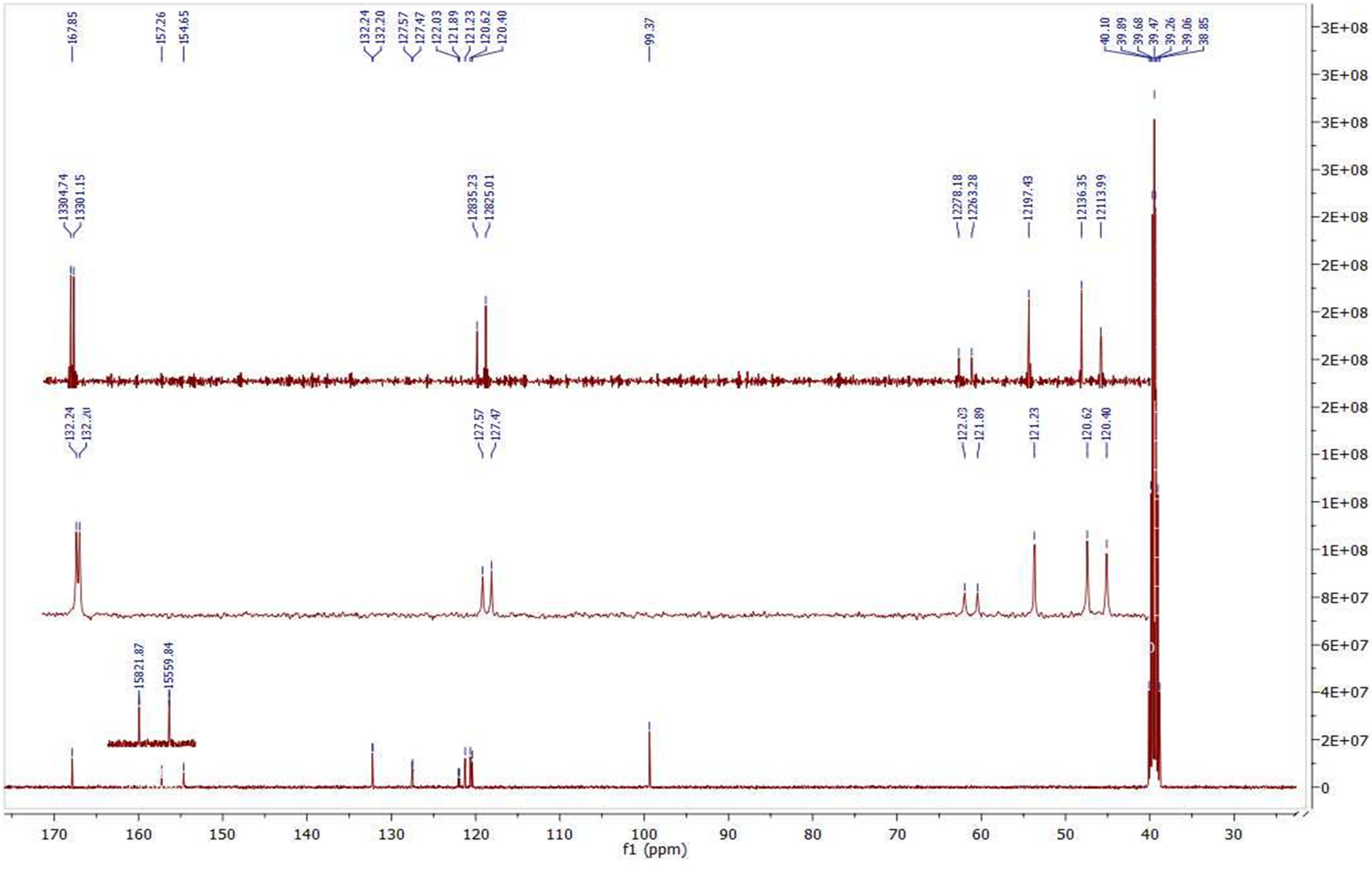

Compound 5a

^{1}H-NMR spectrum of 5a recorded at 300 MHz

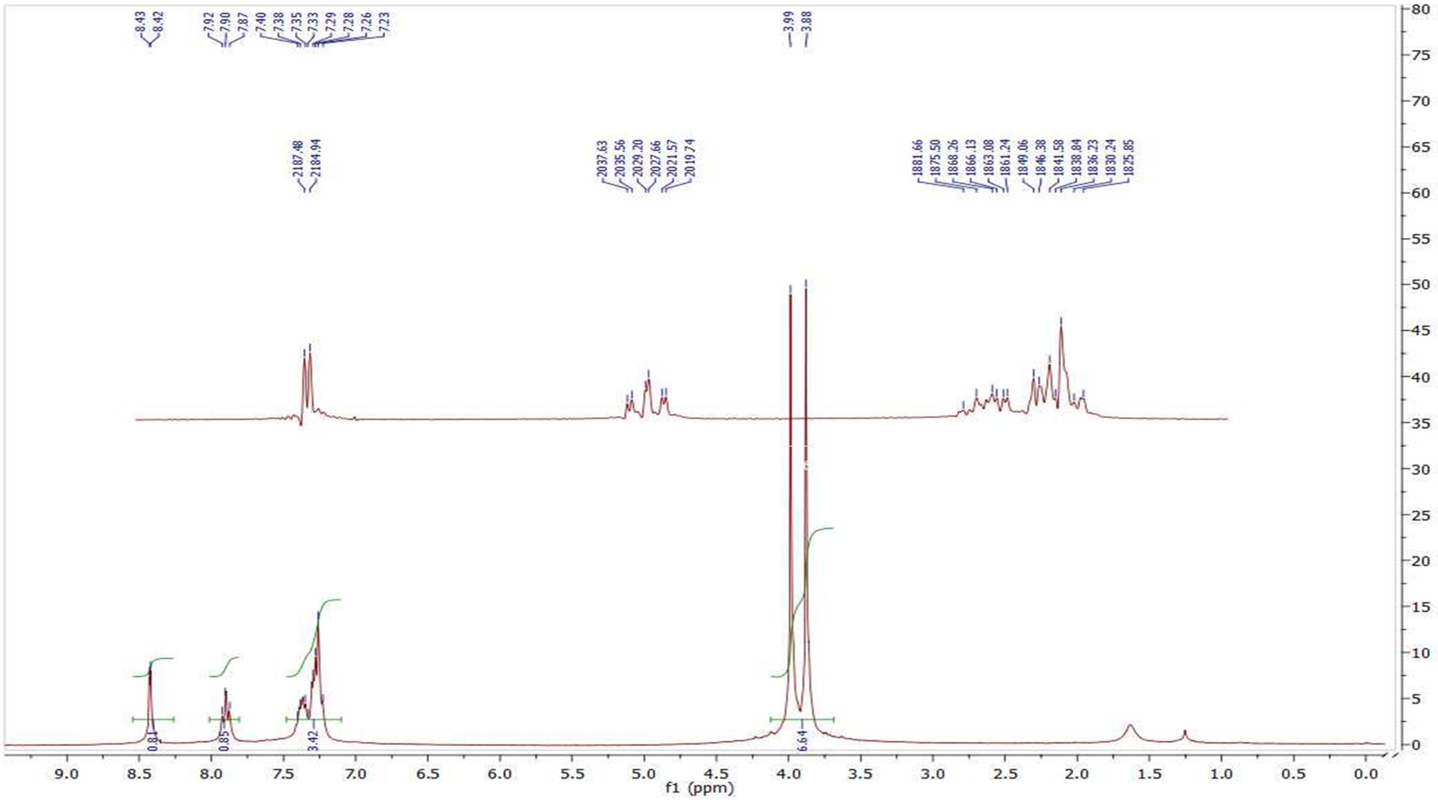

^{13}C-NMR spectrum of 5a recorded at 75 MHz

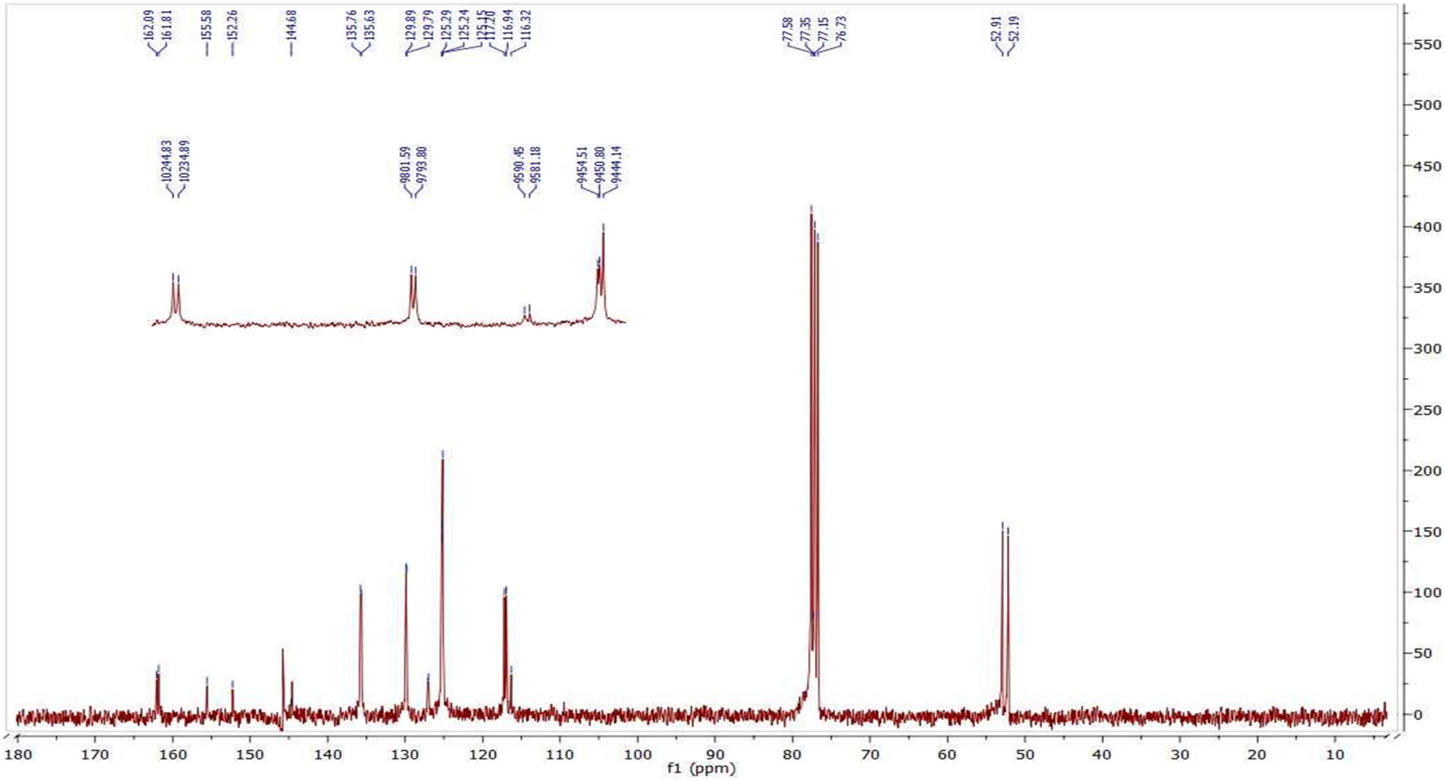

Compound 5b

^{1}H-NMR spectrum of 5b recorded at 300 MHz

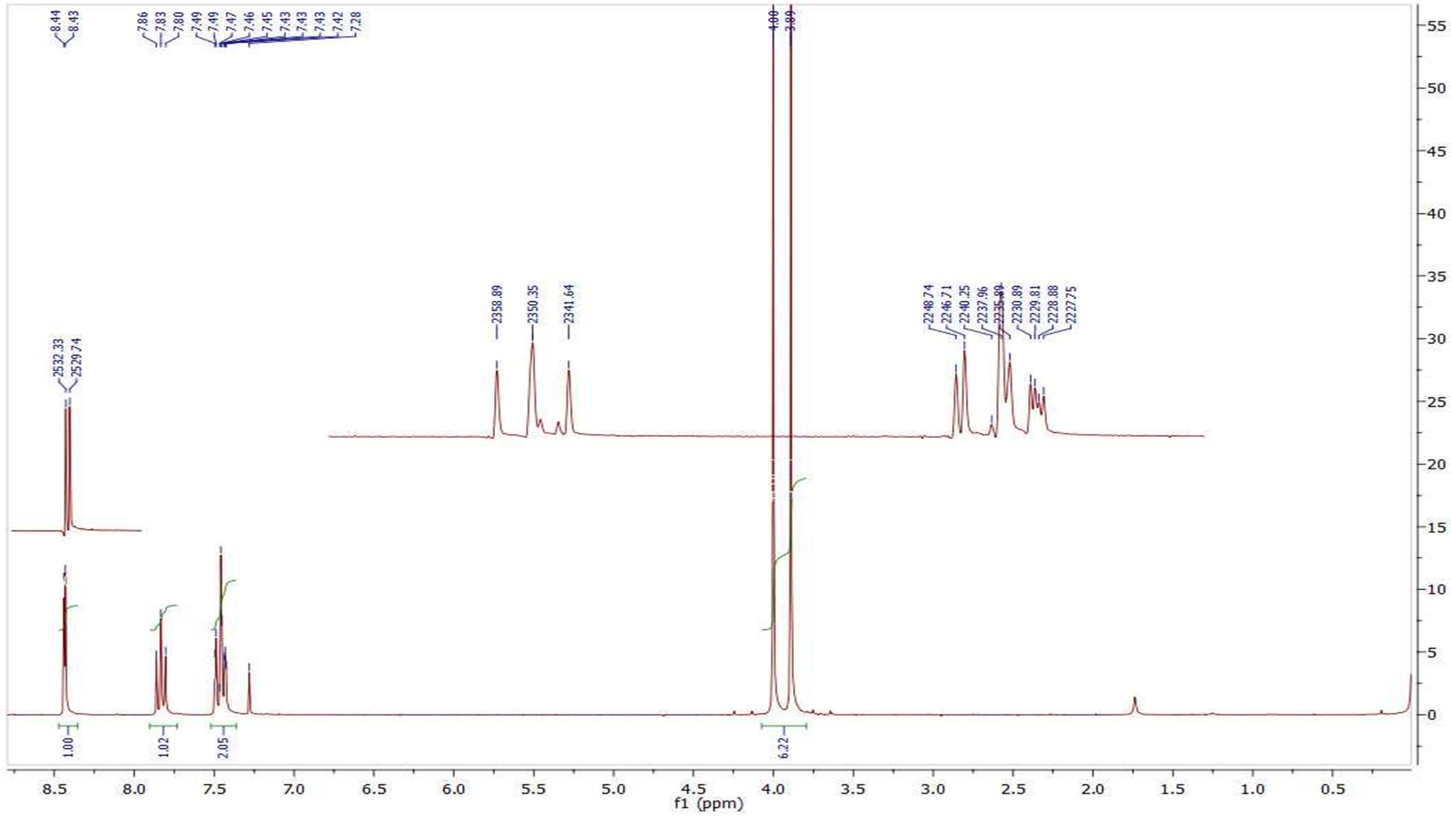

^{13}C-NMR spectrum of 5b recorded at 75 MHz

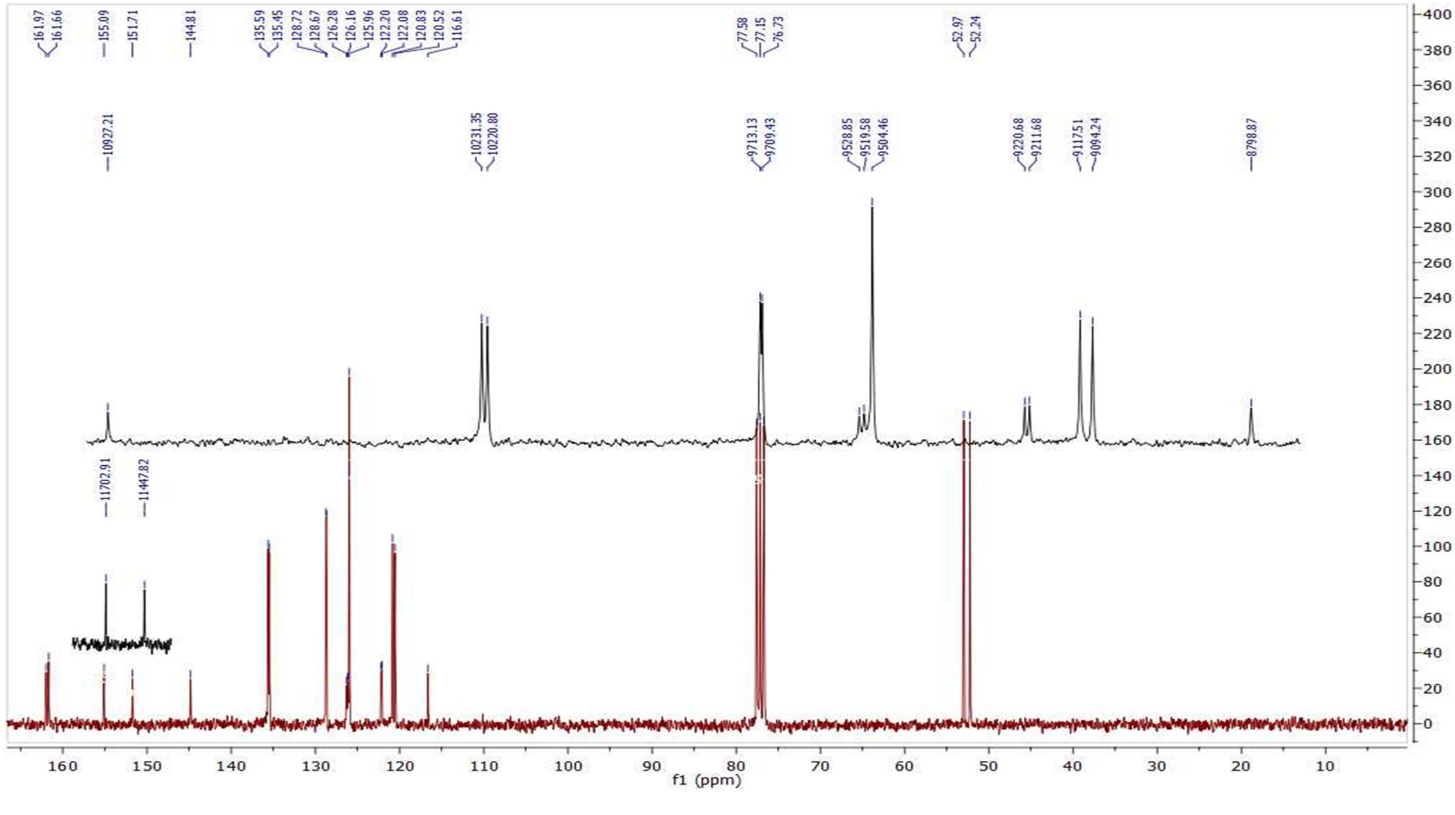

^1H-^1H Correlated Spectroscopy (COSY) of 5b (aromatic protons)

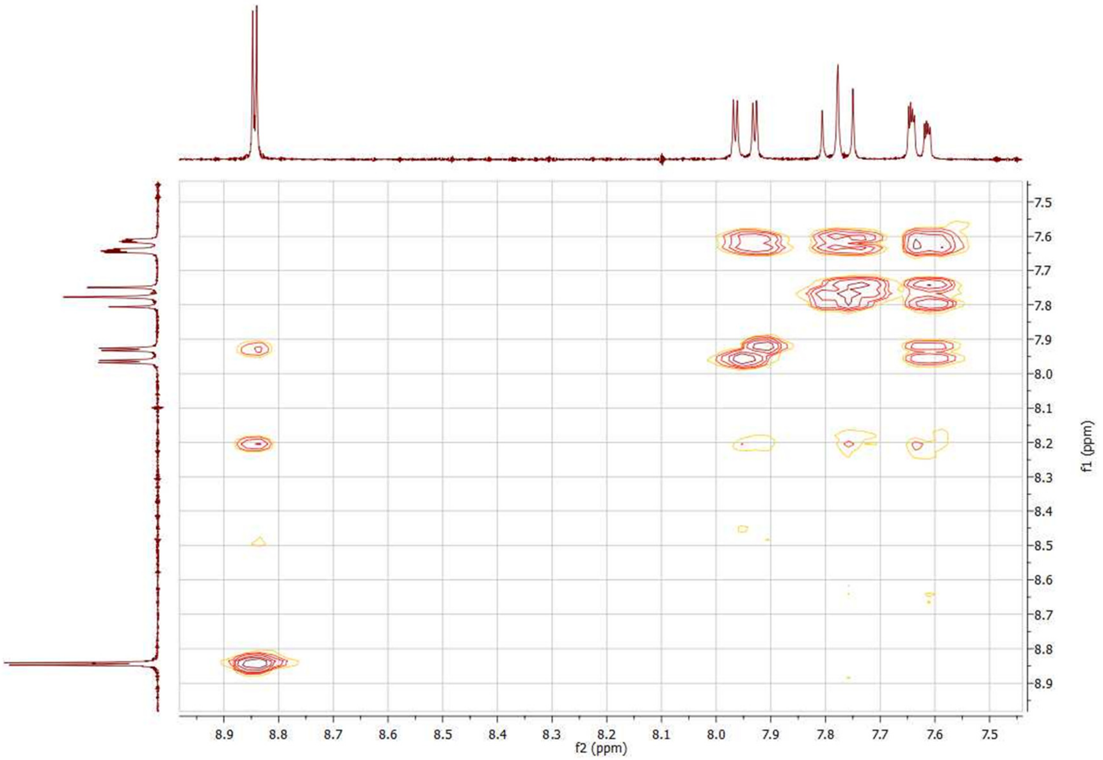

^1H-^{13}C Heteronuclear Correlation spectrum (HETCOR) of 5b (aromatic area)

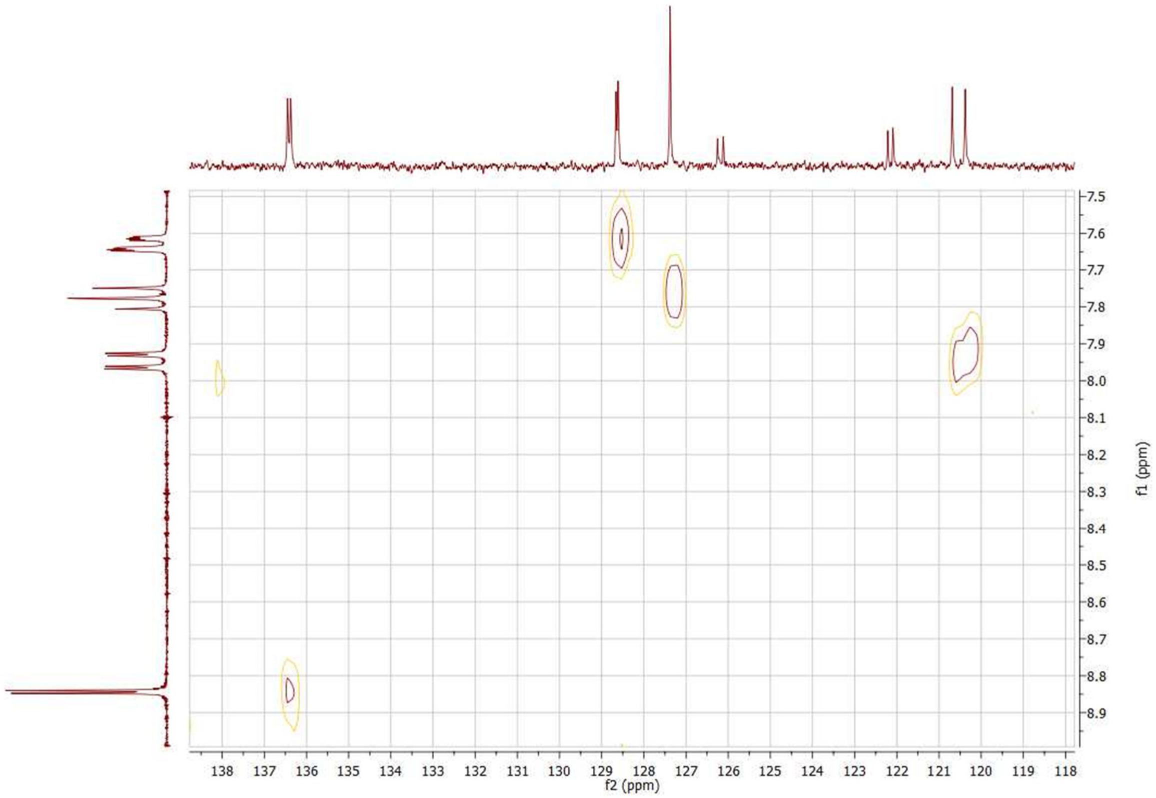

Compound 5c

^{1}H-NMR spectrum of 5c recorded at 300 MHz

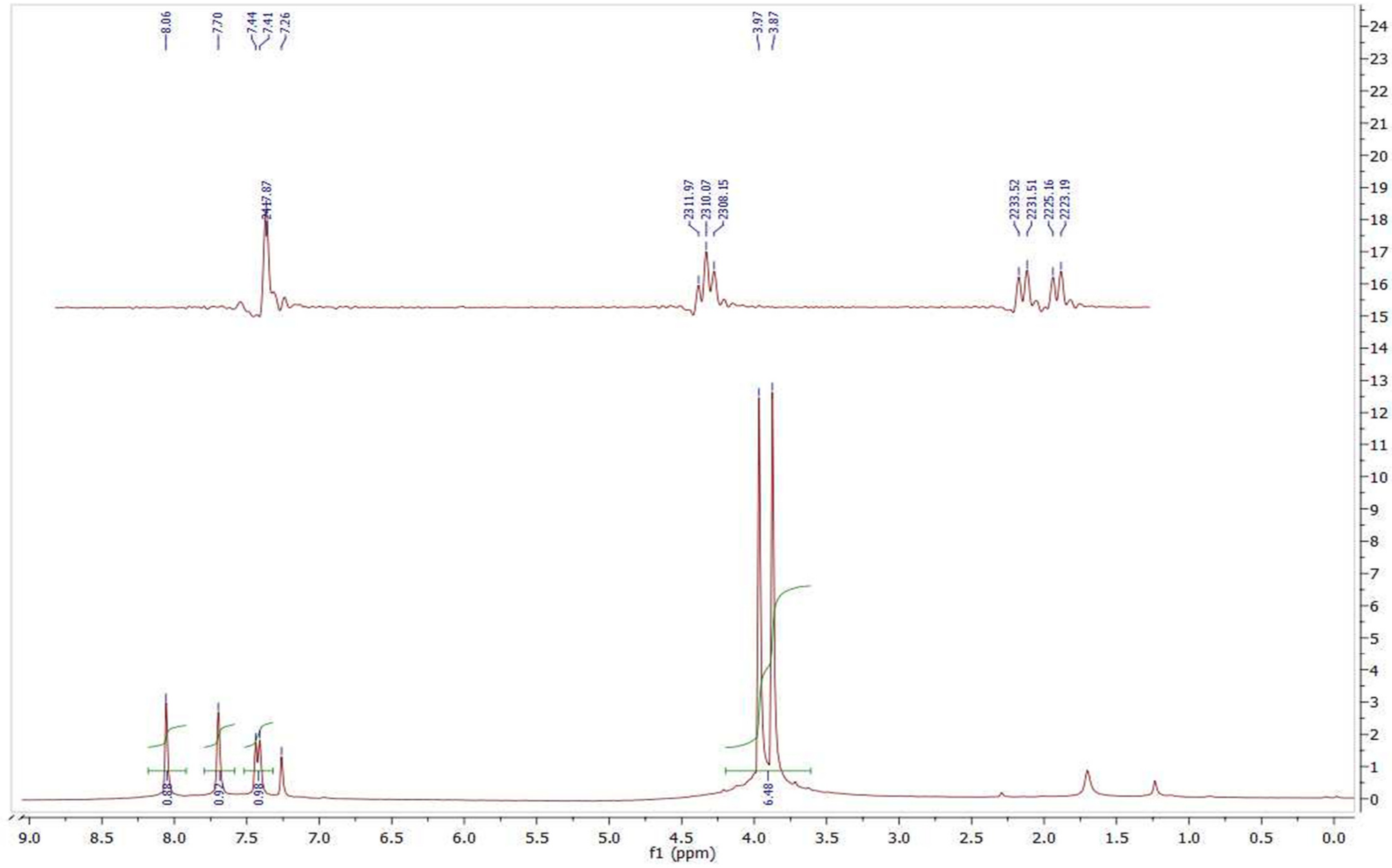

^{13}C-NMR spectrum of 5c recorded at 75 MHz

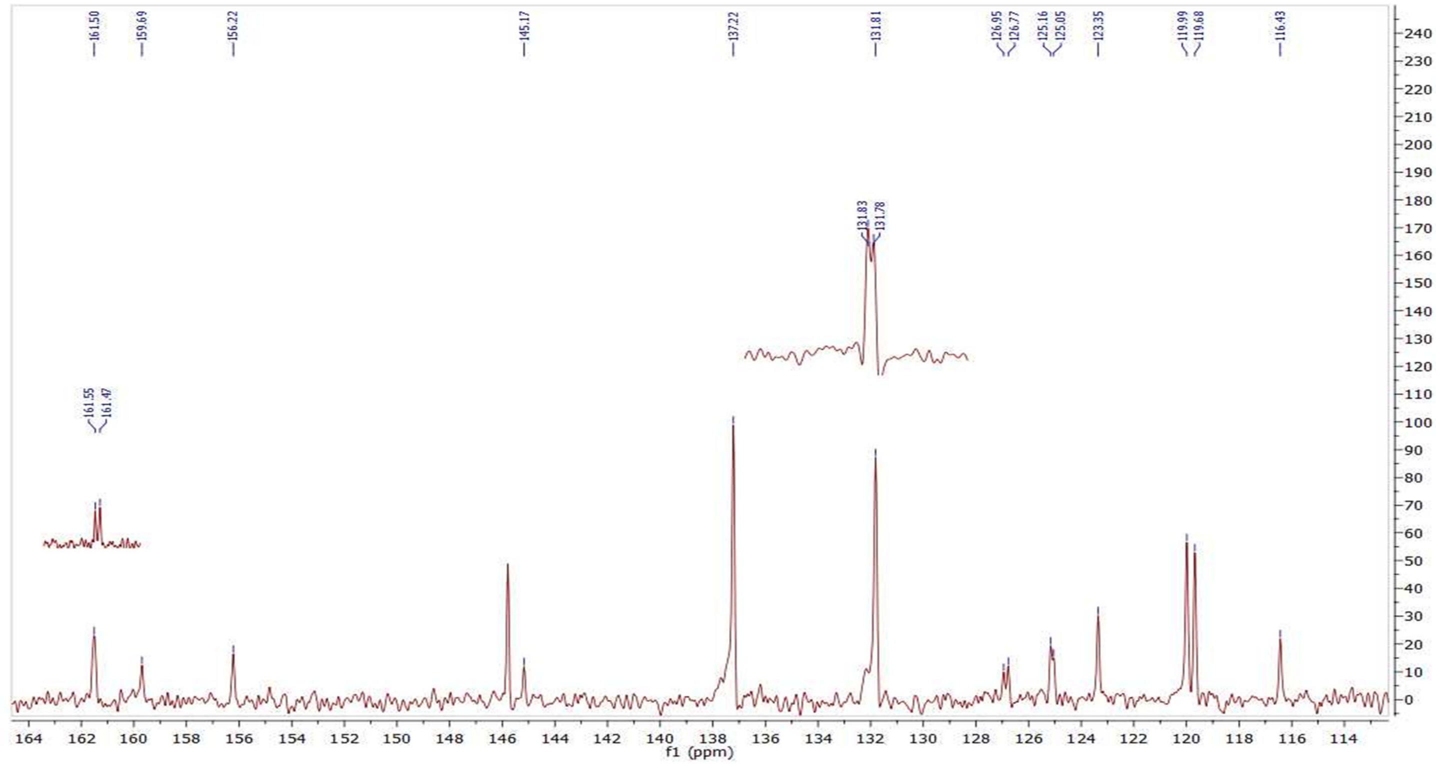

Supplement: Supplementary file 1 [file molecules-26-03693-s001.zip › molecules-1223143-supplementary.pdf]
